# The Effects of Climate Change on Landscape Connectivity and Genetic Clusters in a Small Subtropical and Warm-Temperate Tree

**DOI:** 10.3389/fpls.2021.671336

**Published:** 2021-11-10

**Authors:** Bicai Guan, Jingjing Gao, Wei Chen, Xi Gong, Gang Ge

**Affiliations:** College of Life Sciences, Nanchang University, Nanchang, China

**Keywords:** adaptation, climatic suitability, evolutionary potential, range shift, population connectivity

## Abstract

Climate change is a great threat to global biodiversity and has resulted in serious ecological consequences. Although the potential effects of climate change on genetic diversity have recently received much research attention, little research has focused on the impacts of climate change on genetic connectivity and the relationship between climate stability and genetic divergence. Here, we combined population connectivity with genetic data to predict the impacts of future climate change on genetic connectivity. Coupled with climatic variables and genetic data, we used POPS software to create spatially explicit simulations and predict the dynamics in genetic clusters in response to climate changes. A generalized additive model was employed to test the correlation between climatic stability and genetic diversification. Our findings indicated that a reduction in species distribution due to severe climate change would lead to a substantial loss of genetic connectivity. More severe future climatic scenarios would likely cause greater loss of variability or more distinct homogenization in genetic variation of species. Relatively low interpolated genetic distances are generally associated with areas of greater losses in climatic suitability from the present to the future. The displacement of climatic genetic clusters will challenge species adaptation to future climate change because of the loss of fundamental evolutionary potential. The persistence capacity of plant species may be weakened in the face of future climate change.

## Introduction

Modern reliance on fossil fuels has caused unprecedented climate change, ushering in extreme temperatures globally and abnormal precipitation patterns in many regions (the Intergovernmental Panel on Climate Change, IPCC, 2007; Sheffield and Wood, 2008). These changes have resulted in serious ecological consequences, such as latitudinal and altitudinal shifts in geographic ranges, altered phenology, disrupted physiology, and disturbed community dynamics (Harte and Shaw, 1995; Hickling et al., 2006; Parmesan, 2006; Springer and Ward, 2007; Lenoir et al., 2008; Anderson et al., 2012; Leonardi et al., 2012; Siepielski et al., 2017). Owing to the rapid pace of global change, many species have suffered local population extinctions with low dispersal abilities to track preferred climates, and habitat fragmentation will likely further impede migration (Davis and Shaw, 2001; Kremer et al., 2012), leading, in turn, to potentially further range contraction (Parmesan, 2006; Sinervo et al., 2010; Cahill et al., 2012). Climate change is considered an important factor that threatens species persistence and landscape connectivity (McIntyre et al., 2014). The reduction in population connectivity will be likely to result in a decrease in dispersal (Gibbs, 1998; Wasserman et al., 2012) and reduce population viability and gene flow (Reh and Seitz, 1990; Wilson and Provan, 2003; Wasserman et al., 2012). Therefore, the loss of genetic diversity associated with decreases in landscape connectivity may increase the risk of local population extinctions (Hoffmann and Sgrò, 2011). Thus, landscape connectivity prediction for future global change may provide valuable information for species persistence (Brown and Yoder, 2015; Huerta-Ramos et al., 2015; Inoue and Berg, 2017). Landscape genetics analysis approaches can contribute to predict genetic patterns in response to climatic change (Wasserman et al., 2013; Brown and Yoder, 2015; Johnson et al., 2017; Guan et al., 2019). From a conservation perspective, it is important to identify areas that both harbor high genetic diversity and are projected to remain within a climatically suitable space under future climate change scenarios (Taubmann et al., 2011). Thus, there is a demand to explore the possibility of a mechanistic link between climatic fluctuation and genetic diversity to more completely predict the effects of climate change on genetic variation.

China harbors the most diverse temperate flora of the world, with at least 3,000 vascular plant genera, of which a remarkable 8.2% (c. 248) is endemic (Wu et al., 1996; López-Pujol et al., 2006, 2011). These taxa are concentrated mainly in the subtropic and warm temperate zone across central and southeast China, and covers over 5,000 km from west to east. The region is highly sensitive to global change, which greatly affects endemic species distribution. However, climate change and human activities, such as rapid urbanization, have posed serious threats to biodiversity in China over the past several decades (Li and Chen, 2014; Cui et al., 2016; Sajjad et al., 2018). For instance, the 2008 chilling event in central and southeast China led to the regional extinction of many tree species, with a loss of over 1.86 × 10^7^ hm^2^ of forest (Chen et al., 2012). It was estimated that in terms of area, 40% of ecosystems were degraded severely, 15–20% of species were highly threatened, and genetic diversity suffered greatly from heavy erosion (Ma et al., 2004; Nie et al., 2014). What is more worrying is that projects suggest mainland China might continue to confront particular risks from global warming in the coming decades, with a rapid rise of the annual mean temperature by 2.3–3.3°C and of precipitation by 5–7% by 2050 (Writing Committee of National Assessment Report on Climate Change, 2014). The accelerated warming could negatively affect the ability of plant populations to respond to further climate change (Cao et al., 2016), and is viewed as the greatest threat to the persistence of biodiversity in central and eastern China in the future (Dai et al., 2009; Li X. D. et al., 2011). Although most studies have focused on the impact of climate warming on spatial and phenological shifts of species and genetic diversity, relatively little research has been conducted on the potential effects of climate change on landscape connectivity and the correlation between climatic stability and genetic diversification. Thus, a more comprehensive understanding of the genetic consequences of climate change is required to evaluate the impacts of climate change on landscape connectivity and the relationship between climate stability and genetic divergence.

We chose the small deciduous tree *Cornus kousa* subsp. *chinensis* as our model species. It is broadly distributed across subtropical and warm-temperate deciduous forests of mainland China. The species grows on environmental heterogeneities, such as variation in elevation, precipitation, temperature, moisture, and soil-nutrient composition, occurring from the subtropical zone to the temperate zone across its natural distributional range in central and eastern China (Flora of China Editorial Committee., 2005). The red globose fruit is about 1–1.5 cm in diameter and often falls near the mother plant at maturity. Its main seed dispersers are rodents, which have no ability to spread seeds over long distances (Li N. et al., 2011). Population persistence of the species has a profound significance on the healthy function of forest ecosystems in mountains (Pais et al., 2017). Here, to examine the potential effects of climate change on evolutionary potential, we study how climate change could fragment the genetic landscape and affect genetic clusters. We conduct landscape genetics analyses to test the correlation between variation in the level of genetic diversity and climatic fluctuation and aid our ability to predict the vulnerability of species in the face of future environmental changes.

## Materials and Methods

### Species Distribution Modeling and Landscape Connectivity

**FIGURE 1:**
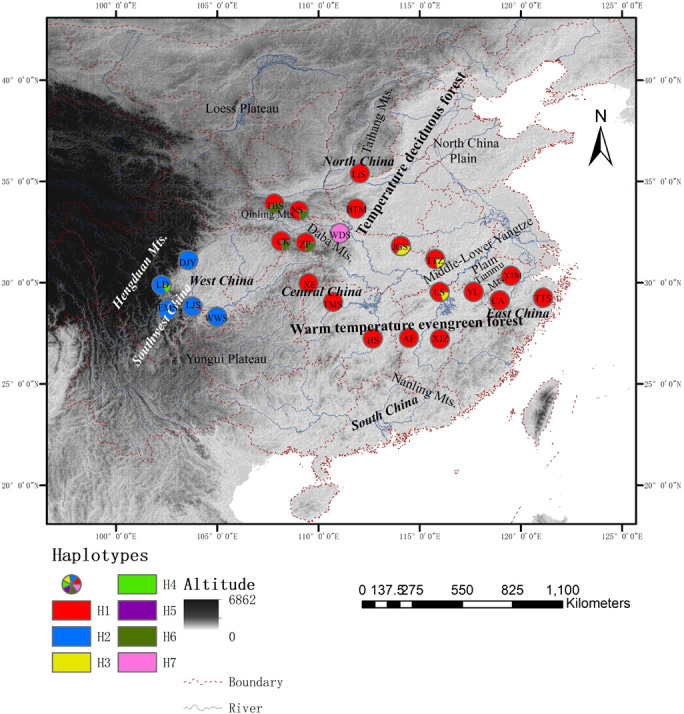
Distribution of shared haplotypes and population locations of *Cornus kousa* subsp. *chinensis*. Different color circles are designated as haplotypes H1∼H7, respectively. Letters in the circles are the acronym of the populations.

Projected climate data layers for the current (∼1950–2000) and the 2080 period (average for 2061–2080) based on 19 bioclimatic variables at a resolution of 2.5 arc-min (ca. 21 km^2^) were downloaded from the WorldClim database. Different general circulation models (GCMs) vary considerably in future projections (Jayasankar et al., 2015). Therefore, we examined three GCMs (CCSM4, HadGEM2-ES, and MIROC-ESM) for future projections of the 2080 period with RCP45 and RCP85. RCP85 denotes the worst-case scenario, which entails the highest projected increase in globally averaged greenhouse gas concentrations, and RCP45 represents the medium-case scenario (Vuuren et al., 2011). Because some climate variables are highly correlated with each other, bioclimatic variables with Pearson correlation above 0.75 were removed to reduce the autocorrelation of input environmental data. To project future distributions, the average of the outputs of three GCMs for each variable was used in building the model to account for variability between model projections. The Digital Elevation Model (DEM) dataset was downloaded from the Cold and Arid Regions Sciences Data Center in Lanzhou, China^1^. The Chinese vegetation (VEG) dataset was obtained from the Environmental and Ecological Science Data Center for West China National Natural Science Foundation of China (see text footnote 1). Land cover data were obtained from the Moderate Resolution Imaging Spectroradiometer (MODIS)^2^. To construct species distribution models (SDMs), we gathered a total of 492 occurrence records of *C. kousa* subsp. *chinensis* covering the entire range known for the species. These occurrences were mainly collected from the IUCN Red List Data^3^, Chinese Virtual Herbarium (CVH)^4^, and previous literature. To ensure optimum SDM performance, the occurrence data must be spatially independent; therefore, the elimination of spatially auto-correlated occurrence points was crucial for model evaluation. Spatially independent occurrences can avoid the prediction over-fitting problem and improve model performance (Veloz, 2009; Hijmans, 2012; Boria et al., 2014). Hence, a method that spatially rarefies localities at specified Euclidian distance was employed according to landscape heterogeneity (Boria et al., 2014). All the occurrence points were rarefied at 25 km^2^ in areas of low climatic heterogeneity. To estimate landscape heterogeneity, we performed a principal component analysis (PCA) for the environmental variables (Guan et al., 2016). Finally, together with the weakly correlated environmental layers, the remaining 129 localities (Supplementary Table 1) with spatially independent occurrence were used to build the SDMs (Figure 1).

The SDMs were constructed with R package Biomod2 (Thuiller et al., 2014). Ensemble modeling techniques allow for the evaluation of the range of projections and enable more reliable predictions. SDMs require species absence point data, which we obtained using pseudo-absences (PAs) methods, to predict suitable species habitat. The distribution of species was modeled using 10 different algorithms: generalized additive models (GAMs), generalized linear models (GLMs), random forest (RF), classification tree analysis (CTA), flexible discriminant analysis (FDA), artificial neural networks (ANNs), surface range envelope (SRE), generalized boosted models (GBMs), multivariate adaptive regression splines (MARSs), and maximum entropy (MaxEnt). The ensemble models were built using the true skill statistic (TSS) and the area under the receiver operating characteristic curve (AUC) for current and 2080 under two greenhouse gas scenarios (RCP45 and RCP85) (Fielding and Bell, 2002; Marzban, 2004; Howell et al., 2011). All the 10 models were constructed with 75% training and 25% testing of occurrence data, which was repeated 10 times for each model. All the models with thresholds of TSS and AUC above 0.6 were retained for ensemble modeling (Guan et al., 2020). Finally, the ensemble models of the distribution of *C. kousa* subsp. *chinensis* were produced with the optimum performance models.

We transformed all the ensemble models into binary SDMs by reclassifying model pixels with the lowest presence threshold (LPT) (Pearson et al., 2007) *via* ArcGIS 10.2. Then, we used the SDM toolbox v1.2 in ArcGIS to calculate the distribution shifts between the current and future species distribution and forecast range expansion, contraction, and stability (Brown, 2014).

### Plant Materials, DNA Isolation, and Sanger Sequencing

Leaf material was obtained from 24 populations (Supplementary Table 2) of *C. kousa* subsp. *chinensis* throughout its distribution across central and eastern China. Whole genomic DNA was extracted from the dried leaf tissue Plant DNA-Easy kit (Bioteke Corporation, Beijing, China). For the DNA survey, two intergenic spacer regions (IGSs) (*rpl*14*-rpl*36 and *trn*L*-trn*F) of chloroplast DNA (cpDNA) were sequenced for all *C. kousa* subsp. *chinensis* samples (*n* = 186). The sequences were edited, assembled, and aligned in GENEIOUS version 4.8.4 (Drummond et al., 2010) after generating with an ABI 377XL DNA sequencer. All the cpDNA haplotype sequences were deposited in GenBank. Accession numbers were generated (see Supplementary Table 3).

Following the protocols specially developed for *Cornus kousa* by Wadl et al. (2010), eight SSR markers (CK007, CK015, CK029, CK031, CK040, CK043, CK047, and CK048) and amplification were used for genotyping 215 samples. The PCR products were purified and then loaded on an ABI 3730XL DNA Analyzer. The data were scored and compiled using GENEMARKER v.2.2.0 (SoftGenetics, State College, PA, United States).

### Evaluating Genetic Data and Parameters

To forecast the impacts of climate change on genetic clusters of *C. kousa* subsp. *chinensis*, we evaluated genetic parameters based on the cpDNA sequences and nuclear microsatellite loci. For the microsatellite loci, linkage disequilibrium (LD) was analyzed using GENEPOP version 4.0.7 (Rousset, 2008). GenAlEx v 6.502 (Peakall and Smouse, 2012) was employed to calculate the value of Shannon’s Information Index (I), the number of effective alleles (*N*_*e*_); expected heterozygosity (*H*_*e*_) under Hardy–Weinberg equilibrium following Nei (1978) for each population. To avoid underestimating *N*_*e*_, we removed populations with sample sizes less than 5 (Table 1). For sequence data, we calculated the nucleotide diversity (π_*T*_), gene diversity (*h*), and the number of haplotypes (*N*_*h*_) (Nei, 1987) for each population. The analyses were performed with the software DnaSP 5.10 (Librado and Rozas, 2009).

**TABLE 1:** Genetic diversity of the 24 populations of *Cornus kousa* subsp. *chinensis* for microsatellite loci and chloroplast sequences.

	Microsatellites				Sequences				Suitability		
Pop	*N*	I	*N*_*e*_	*H*_*e*_	*N*	*h*	π_*T*_	N_*h*_	Current	Future RCP45	Future RCP85
AF	6	0.79	2.2	0.450	4	0.5	0.0037	2	0.358	0.391	0.461
BTM	14	1.12	3.0	0.559	8	0.25	0.0018	2	0.701	0.623	0.505
CA	5	1.24	3.5	0.653	6	0.33	0.0003	2	0.927	0.969	0.923
CK	5	1.2	3.3	0.635	9	0.22	0.00016	2	0.875	0.447	0.513
DJY	7	0.87	2.4	0.491	8	0.25	0.0018	2	0.666	0.310	0.532
EMS	15	0.84	2.6	0.441	6	0.33	0.0002	2	0.571	0.502	0.394
HS	10	1.09	3.0	0.557	12	0.30	0.0002	2	0.813	0.854	0.692
JGS	9	1.27	3.9	0.606	12	0.35	0.0004	2	0.850	0.442	0.688
LD	10	1.10	2.7	0.554	6	0.6	0.0029	2	0.466	0.240	0.220
LS	5	1.28	3.7	0.658	10	0	0	1	0.946	0.970	0.887
LiS	5	1.08	3.0	0.605	4	0	0	1	0.896	0.372	0.289
NS	7	0.91	2.6	0.491	9	0.57	0.0004	2	0.950	0.746	0.550
TBS	10	1.13	2.8	0.602	8	0.57	0.0054	2	0.600	0.587	0.457
TMS	11	1.18	3.2	0.612	9	0.5	0.0004	2	0.955	0.894	0.819
TTS	5	0.87	2.7	0.504	4	0.67	0.0005	2	0.848	0.944	0.785
TTZ	11	1.33	3.8	0.645	10	0.47	0.0003	2	0.968	0.873	0.875
WDS	5	0.79	2.1	0.450	4	0	0	1	0.904	0.768	0.545
XE	16	1.06	3.0	0.556	11	0.33	0.0002	2	0.920	0.899	0.795
XJZ	10	1.56	4.9	0.714	4	0.67	0.0005	2	0.832	0.931	0.766
XTM	7	1.10	3.1	0.557	7	0.57	0.0004	2	0.828	0.942	0.831
YL	11	1.05	3.3	0.509	9	0.22	0.0002	2	0.934	0.972	0.853
ZP	5	1.15	3.2	0.617	4	0	0	1	0.534	0.617	0.558
LJS	13	0.77	2.0	0.458	15	0.13	0.0039	2	0.432	0.234	0.376
WWS	13	0.45	1.6	0.286	10	0	0	1	0.330	0.203	0.232

*Pop, population; N, number of individuals; I, Shannon’s Information Index; N_e_, number of effective alleles; H_e_, expected heterozygosity; h, gene diversity; π_T_, nucleotide diversity; N_h_, number of haplotypes. Suitability represents the environmental suitability obtained by species distribution modeling.*

We assumed that a displacement of climatically suitable habitats for the species in a geographical space will occur in response to climate change and, consequently, that only populations located in areas above the lowest presence threshold (LPT) of habitat suitability will persist and generate the gene pool of individuals for the next generation. To predict the genetic diversity of *C. kousa* subsp. *chinensis* in the future, we recalculated the genetic parameters of diversity mentioned above using LPT for both future climate change scenarios (RCP45 and RCP85). For nuclear microsatellites, the simulations were executed for each locus and overall loci with the software GenAlex 6.502 (Peakall and Smouse, 2012), whereas for sequence data, we performed data simulation with DnaSP 5.10 (Librado and Rozas, 2009).

### Modeling the Landscape Connectivity

To determine whether environmental suitability is responsible for shaping patterns of genetic connectivity among populations, we performed Mantel and partial Mantel tests with software Zt version 1.1 (Bonnet and Van de Peer, 2002). Genetic divergence was calculated in the ARLEQUIN v3.5 package (Excoffier et al., 2005) based on linearized *F*_*st*_ values [*F*_*st*_/(1−*F*_*st*_)]. Landscape resistance values were exacted from a friction layer from inverted SDMs. Then, the matrices were generated with GenAlEx v 6.5 (Peakall and Smouse, 2012). Genetic distance matrices were correlated against the landscape resistance matrices with competing for Euclidian distance partialed-out in partial Mantel tests.

Ecological dispersal networks for species were created by applying categories of the least-cost paths (LCPs) method, which computed the least dispersal costs between two localities. In this approach, we inverted the SDMs produced by Biomod2 to obtain a friction layer (dispersal cost layer) for the current and future (2080) periods. That is, areas of high suitability had a low dispersal cost through the landscape, whereas regions with low or no probability of occurrence were converted to areas of high dispersal costs. Subsequently, we used the friction layer to generate a cost distance layer for each locality. Finally, based on the cost distance layer, we created pairwise populations and shared a cpDNA haplotype network by adding the LCPs between two populations. The models were generated using SDM toolbox version 1.2 in ArcMap 10.2 (Brown, 2014).

### Predicting Dynamics in Genetic Clusters Under Future Scenarios

We used the POPS software (Jay et al., 2015) to execute spatially explicit simulations in order to predict dynamics in genetic clusters in response to climate change (Jay et al., 2012). The software carries out Bayesian clustering algorithms based on genetic, geographical, and environmental variables, and assigns individuals or genes to different genetic groups after simulating the impacts of environmental variables on individuals and admixture proportions. The three climate variables (annual mean temperature, precipitation in the driest month, and annual precipitation) with higher variance among populations were selected to model the effect on genetic clusters. Combining climatic variables and genetic data, we launched POPS to simulate genetic clusters under current climatic conditions (Jay et al., 2015) with the Markov chain Monte Carlo (MCMC) estimation algorithm. The run used 20,000 sweeps following a burn-in period of 2,000 sweeps using models with admixture. The simulations were implemented four times for each value of the maximum number of cluster K ranging between 2 and 20. We selected a subset of runs minimizing the deviance information criterion (DIC, Spiegelhalter et al., 2002). Only the runs with the lowest DIC values were retained; other runs were discarded. We then predicted the genetic clusters for the RCP45 and RCP85 scenarios modeling genetic coancestry in response to future climate change. Finally, we computed correlation coefficients measuring the relationship between matrices of current and projected ancestry coefficients for the RCP45 and RCP85 scenarios. The correlation facilitates understanding of the effects of climate change on spatial genetic structure. The closer the correlation is to 1, the smaller the expected changes in spatial genetic structure.

### Examining the Relationship Between Genetic Diversity and Climatic Stability

We used the Genetic Landscapes Toolbox (Vandergast et al., 2011) to generate divergence landscapes in ArcGIS 10.2. Pairwise genetic distance was visualized as genetic landscapes and mapped to the geographic midpoints between collection locations with the Single Species Genetic Divergence Tool. A continuous layer was interpolated from the geographic midpoints with Inverse Distance Weighted (IDW) interpolation in grid cell size 1 km^2^ (power = 2, variable search radius with 12 points). To avoid extrapolating beyond the original sample locations, the raster was clipped to the spatial extent of sample locations. High values represent areas of high genetic differentiation between sampled locations, and low values mean relatively low genetic divergence between collection locations. A generalized additive model (GAM) was used in R 3.6.1 to test the correlation between climatic stability and genetic diversification. Interpolated genetic distance was used as the response variable, and change between the present and the future was used as the predictor variable. These values were calculated by extracting the value of the difference between the current and present climatic suitability at each point at which an interpolated genetic distance was calculated.

## Results

### Genetic Data and Parameters

The total alignment of the two chloroplast regions (*trnL-trnF* and *rpl14-rpl36*) was surveyed across the 186 individuals of *C. kousa* subsp. *chinensis* was 1,566 bp in length, including four indels and 31 substitutions. Together, all the polymorphisms identified seven chloroplast haplotypes (chlorotypes, H1–7) (Supplementary Table 3) across the 24 populations (Figure 1). At the species level, the cpDNA data revealed that nucleotide diversity (π_*T*_) was 1.08 × 10^–3^ and that haplotype diversity (H_*d*_) was 0.447. Microsatellite data were obtained across the 215 individuals of *C. kousa* subsp. *chinensis* genotyped at eight microsatellite loci (Table 1 and Figure 1). All eight microsatellite loci displayed polymorphism. On average, *H*_*e*_, *N*_*e*_, and I were 0.55 (range: 0.29–0.714), 2.98 (range: 1.6–4.9), and 1.05 (range: 0.45–1.56), respectively (Table 1). There was no significant linkage disequilibrium (LD) detected between any pair of loci.

### Species Distribution Models and Landscape Connectivity

All the ensemble forecasting models had an AUC and a TSS above 0.6, indicating the effectiveness of these in distinguishing suitable and unsuitable habitats. The mean AUC and TSS values for 10 algorithms calculated in the models are listed in Supplementary Table 4. The presence probability of *C. kousa* subsp. *Chinensis* was highly related to annual mean temperature and precipitation in the driest month (Supplementary Table 5), whereas the importance of land cover and vegetation affecting the SDMs was very low. The annual mean temperature was the most important environmental variable affecting species distribution, from 0.351 to 0.964 with a mean of 0.592. Precipitation in the driest month had importance from 0.239 to 0.798 with a mean of 0.419.

**FIGURE 2:**
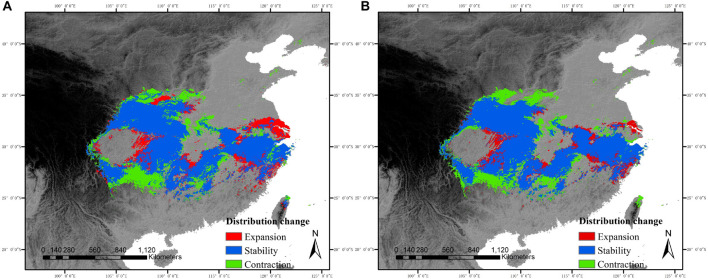
Range shifts of *C. kousa* subsp. *chinensis* in relation to time intervals from the current period to the year 2080. The areas with red, blue, and green colors designate range expansion, range stability, and range contraction, respectively. **(A)** RCP45 scenario. **(B)** RCP85 scenario.

The SDMs showed high levels of suitability for *C. kousa* subsp. *chinensis* throughout most of central China and parts of eastern China, except for the Sichuan Basin and middle-lower Yangtze plain. For future scenarios, the species was likely to lose its climatic suitability at most of its ranges, especially the west of its range. By comparing the species ranges under the two future scenarios, we predicted that the effect of climate change on species ranges under the RCP85 scenario would be more drastic than that under the RCP45 scenario. Under the future scenarios, species ranges contracted in the west and expanded in the east (Figures 2A,B), while the species showed a main range contraction as a whole. Range sizes of *C. kousa* subsp. *chinensis* were predicted to decrease by 127,513 km^2^, a reduction of about 15.56%, under the RCP85 scenario, and by 78,689.5 km^2^, a reduction of about 9.05%, under RCP45. Our findings also predicted larger range size changes between current conditions and the RCP85 scenario than RCP45 (Figures 2A,B).

Mantel tests revealed that landscape resistance during the present and future periods were both statistically significantly correlated with genetic distance within a 95% confidence interval. The correlation values of the present, RCP45, and RCP85 were 0.359, 0.345, and 0.389, respectively. The correlation coefficient of 0.16 between Euclidian distance and genetic distance was not significantly different from zero with a *p*-value of 0.119. Partial mantel tests showed that there was a significant correlation between landscape resistance and genetic distance, independently from geographical distances. The correlation values were 0.427,0.346, and 0.479, with an associated *p*-value smaller than 0.05.

**FIGURE 3:**
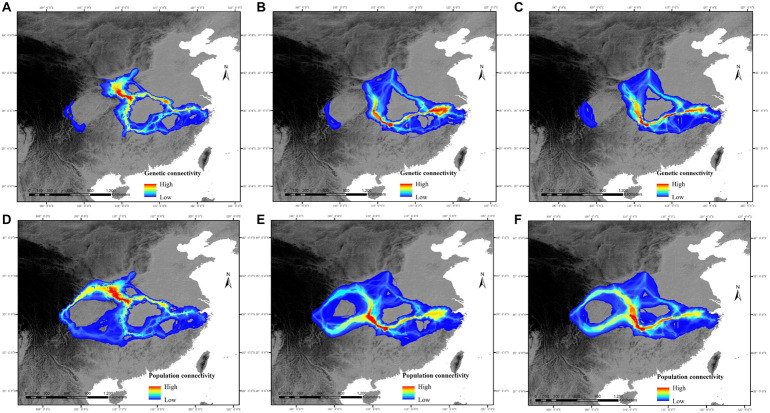
Potential genetic connectivity and population connectivity of *C. kousa* subsp. *chinensis* in relation to two general time periods [the present (∼1950–2000) and the year 2080]. **(A–C)** represent the genetic connectivity of the current period, RCP45 scenario, and RCP85 scenario. **(D–F)** represent the population connectivity of the current period, RCP45 scenario, and RCP85 scenario.

The predictions showed that the five western populations (DJY, LD, EMS, LJS, and WWS) had no genetic connectivity with the central and eastern populations from the present to the year 2080 (Figure 3). Although a little increase in genetic connectivity was predicted for the potential dispersal network among the eastern populations (YL, CA, and XTM), the decline in genetic connectivity was likely to occur among most other populations, especially among the populations distributed in Qingling and Daba Mts. Under the climate warming scenario, LCP models of pairwise populations in central and eastern China revealed similar population connectivity patterns with genetic connectivity patterns (Figure 3). The difference is that the low population connectivity exists between five western populations and other populations in central and eastern China from the present to the year 2080.

### Dynamics in Genetic Clusters in Future Scenarios

**FIGURE 4:**
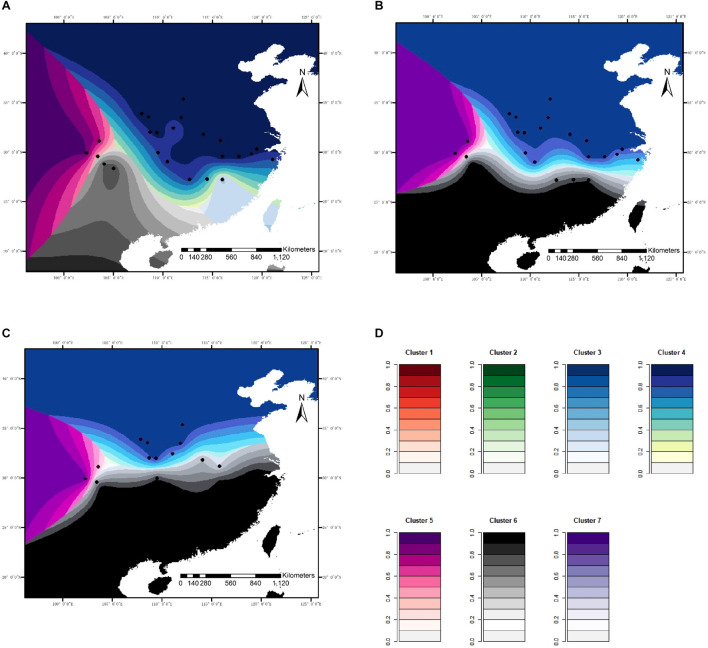
Spatial distribution of genetic clusters in response to climatic variables modeled for *C. kousa* subsp. *chinensis*, for microsatellite loci. **(A)** Current; **(B)** RCP45 scenario; **(C)** RCP85 scenario. **(D)** Each color set corresponds to a cluster in the figure legend. Black dots designate the 24 sampled populations.

**FIGURE 5:**
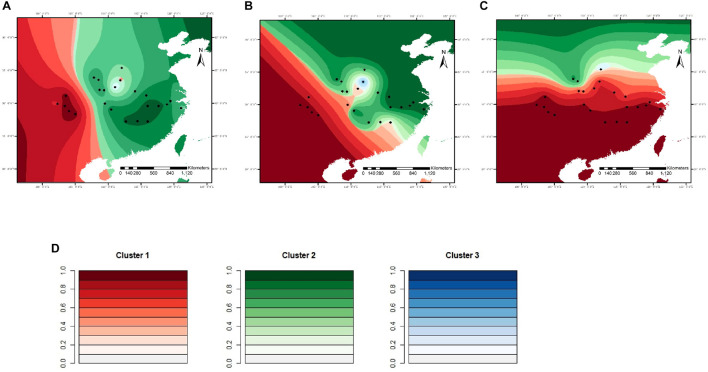
Spatial distribution of genetic clusters in response to climatic variables simulated for *C. kousa* subsp. *chinensis*, for sequence data. **(A)** Current; **(B)** RCP45 scenario; **(C)** RCP85 scenario. **(D)** Each color set corresponds to a cluster in the figure legend. Black dots designate the 24 sampled populations.

**FIGURE 6:**
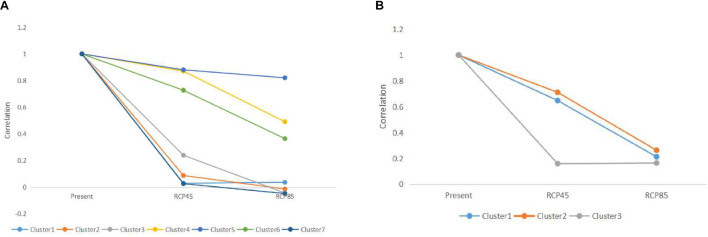
**(A)** Changes in ancestry for the seven genetic clusters simulated with microsatellite data. **(B)** Changes in ancestry for the three genetic clusters simulated with DNA sequence data.

For microsatellite data, the optimal number of genetic clusters in response to climatic variables was *K* = 7 with a DIC value of 9,676 (Supplementary Tables 6–8), the most likely number of genetic clusters was *K* = 3 with a DIC of 709 for sequence data (Supplementary Tables 9, 10). The correlation was computed between the inferred admixture coefficients and those predicted from future climatic covariates. The correlation values of 0.96 for microsatellites and 0.92 for sequence data indicated that the forecasts from the climatic variables were very accurate. Spatially explicit simulations presented loss of variation in climatic clusters due to climate changes (Figures 4, 5), especially under the RCP85 scenario. For microsatellites, climatic clusters are lost in both future scenarios, resulting in homogenization of genetic diversity, such as clusters 2 and 7 for RCP45 (Figure 4B) and 1, 2, and 7 for RCP85 (Figure 4C). Moreover, most populations at the central range of *C. kousa* subsp. *chinensis* were assigned to a common climatic genetic cluster (Figure 4C) because of climate change. For sequence data, cluster 3 is lost for the RCP85 scenario (Figure 5C), while all the clusters were retained under the RCP45 scenario. Moreover, climate change leads to homogenization of genetic variability under both scenarios RCP45 (Figure 5B) and RCP85 (Figure 5C). The simulations showed that genetic variations were more likely to be homogenized under RCP85 than under RCP45 regardless of molecular markers. A turnover analysis also demonstrated that climate change strongly affected the spatial genetic structure of *C. kousa* subsp. *chinensis*; however, different genetic clusters presented varying responses (Figure 6). For microsatellite data, clusters 1, 2, 3, and 7 dropped drastically in ancestry for both future climate scenarios (Figure 6A), whereas the correlation remained larger than 50% in clusters 4 and 5, and cluster 6 showed a moderate shift. For DNA sequences, all three clusters presented significant declines in ancestry under the RCP85 scenario (Figure 6B). However, the correlation cores remained larger than 60% in clusters 1 and 2 under the RCP45 scenario. On the whole, all the genetic clusters presented a higher variation in ancestry for RCP85 than for RCP45.

### Testing the Relationship Between Genetic Diversity and Climatic Stability

**FIGURE 7:**
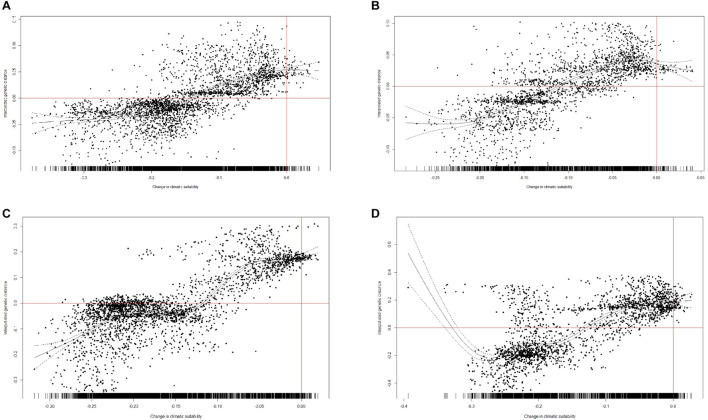
Changes in climatic suitability between the present and the year 2080. **(A)** Threshold = 0.289 (LTP), under RCP85. **(B)** Threshold = 0.6, under RCP85. **(C)** Threshold = 0.289 (LTP), under RCP45. **(D)** Threshold = 0.6, under RCP45. Plotted against interpolated genetic distances at each point at which a value was interpolated.

We performed the GAM analysis of all interpolated genetic distance values in R 3.6.1. The results reveal that areas expected to experience future climate stability are relatively high in genetic diversity and those areas expected to experience future climate fluctuation are relatively low in genetic diversity. The areas expected to lose suitability under future climate change predicted for 2080 in both scenarios are of relatively low interpolated genetic distances, while the regions expected to gain most climatic stability are high in interpolated genetic distances (*R*^2^ = 0.508, *p* < 0.001, threshold = 0.289, under RCP85, Figure 7A; *R*^2^ = 0.571, *p* < 0.001, threshold = 0.6, under RCP85, Figure 7B; *R*^2^ = 0.642, *p* < 0.001, threshold = 0.289, under RCP45, Figure 7C; *R*^2^ = 0.547, *p* < 0.001, threshold = 0.6, under RCP45, Figure 7D). To summarize, relatively high genetic diversity was associated with lower loss in suitability between the future and the present, and relatively low genetic diversity was generally associated with regions of climatic instability between the future and the present.

## Discussion

### Prediction of Range Shifts

Under both climate scenarios, the distributed range size in central and western China may decline, while the distributed areas in eastern China may increase a little (Figure 2) where coastal natural areas were lost to land reclamation and infrastructure construction between 1950 and 2014 because of economic development and rapid urbanization (Cui et al., 2016). Although previous studies have reported that 52 of the 65 plant taxa in central and eastern China have experienced northward range shifts with an average of 3.37° over the past three decades, correlating with recent climate changes (Song et al., 2016), our results predict that *C. kousa* subsp. *chinensis* may expand to eastern China under both future scenarios examined here. Such contrasting results may be caused by the strong variation among plant species, as different species may not respond to climate change in the same way. In addition, the inconsistent findings of this study compared with earlier studies may stem from the research methods used. Previous studies analyzed the range changes of these species by comparing the original occurrence records; for this project, we constructed the models using 3 AOGCM and 10 SDM algorithms in an ensemble method (Araujo and New, 2007; Diniz-Filho et al., 2009), which is considered more reliable than other approaches (Gama et al., 2017).

### Landscape Connectivity and Genetic Diversity

Because of the rapid pace of global change, species will not have the dispersal abilities to track preferred climates, and landscape fragmentation will likely further impede migration (Davis and Shaw, 2001; Kremer et al., 2012). The landscape connectivity of *C. kousa* subsp. *chinensis* may decline in most areas, with the exception of eastern China. The high levels of fragmentation and landscape connectivity decline among populations may hamper population migration and persistence. Thus, the persistence of *C. kousa* subsp. *chinensis* under the climate change scenarios discussed in this study may be threatened despite a little increase of distribution area in eastern China. The shrinkage of the landscape connectivity between central and eastern China may lead to a reduction in effective population size and then result in disruption of the genetic process. Moreover, a decline in genetic connectivity would limit gene flow among populations and cause genetic drift. The species with a decrease in genetic connectivity would face a risk of reduction in adaptative genetic variation, which represents fundamental evolutionary potential. Great reductions in genetic connectivity may impede the adaptation of species to ongoing climate change, raising the risk of local population extinction (Willis et al., 2008; Wright et al., 2008; Taubmann et al., 2011). Having no genetic connectivity with central and eastern populations, western populations may experience genetic drift and then a reduction in evolutionary potential.

### Potential Impact of Climate Change on Genetic Clusters and Genetic Diversity

*Cornus kousa* subsp. *chinensis* prefers to grow in warm, shaded, and moist places. IPCC expert projections forecast that environmental conditions may change drastically during the following decades. The annual mean temperature is predicted to rise by 2.5°C, and precipitation is also likely to increase under the RCP85 scenario in most parts of China, while precipitation in the driest month is predicted to decline remarkably in eastern and southern China in the coming decades (Xu et al., 2006). This will result in spatial transformation of climatic conditions, which will lead climatic clusters to disappear under both future climate scenarios examined here, and shift adaptive landscapes (Wang et al., 2010). Loss of climatic clusters in future scenarios, particularly in the RCP85 scenario, will cause homogenization of genetic diversity. Therefore, we forecast that changes in these climate variables would affect the genetic diversity of species, and could dramatically reduce their fitness. Our prediction is supported by the results of genetic simulations demonstrating a loss of variation among populations and climatic genetic clusters under both future climate scenarios discussed in this project, especially the RCP85 scenario. Most populations from central China are predicted to have shared genetic ancestry as individuals are assigned to the same climatic genetic cluster because of the expectation that annual mean temperatures will rise in these areas. Moreover, a more severe future climatic scenario would be likely to cause greater loss of variation or more distinct homogenization in the genetic diversity of species and, therefore, reduce their fitness. Although preferred to track their ancestral climatic regime under future climate changes, populations with no dispersal abilities (Li N. et al., 2011) must adapt and alter environmental tolerance to respond to varying climatic conditions (Wiens and Graham, 2005). However, adaptation may be insufficient when individuals face severe future climate change (Anderson et al., 2012). Therefore, because of the decline in evolutionary potential, *C. kousa* subsp. *chinensis* may be at risk of local population extinction under future climate change.

Our predictions show areas projected to experience stable climate in the future will overlap with areas of high genetic divergence. Although other factors unrelated to climates, such as land use and habitat fragmentation, are likely to drive variation in levels of genetic diversity and genetic patterns across a distribution of species; this positive correlation can still contribute to identifying areas of high genetic value and high risk of local extinction due to losses in climatic suitability, and enhance our ability to further predict the evolutionary potential of species and local population persistence under future climate change.

## Conclusion

By combining landscape connectivity predictions with genetic simulations, the study shows how to forecast the genetic vulnerability of species and local populations’ persistence in the face of future environmental change. Our findings indicate that the accelerated climatic changes we currently confront may result in the range contraction of *C. kousa* subsp. *chinensis* and affect the genetic connectivity across the landscape, and could potentially lead to a great loss of genetic variation. The displacement of climatic genetic clusters will challenge species adaptation to new environmental conditions because of the loss of fundamental evolutionary potential under climate change.

## Data Availability Statement

The datasets presented in this study can be found in online repositories. The names of the repository/repositories and accession number(s) can be found in the article/Supplementary Material.

## Author Contributions

BG: conceptualization, supervision, funding acquisition, writing the review and editing, sample collection, and data curation. JG: writing – original draft. WC: field investigation, leaf sample collection, and data curation. XG: formal analysis, software, and writing the review and editing. GG: revising the manuscript. BG and GG: ensuring that the descriptions are accurate and agreed upon by all other authors. All authors contributed to the article and approved the submitted version.

## Conflict of Interest

The authors declare that the research was conducted in the absence of any commercial or financial relationships that could be construed as a potential conflict of interest.

## Publisher’s Note

All claims expressed in this article are solely those of the authors and do not necessarily represent those of their affiliated organizations, or those of the publisher, the editors and the reviewers. Any product that may be evaluated in this article, or claim that may be made by its manufacturer, is not guaranteed or endorsed by the publisher.

## Footnotes

1
http://www.ncdc.ac.cn

2
https://lpdaac.usgs.gov/products/modis_products_table/mcd12q1

3
http://www.iucnredlist.org/

4
http://www.cvh.ac.cn
