# Supplementary material for: The Effects of Climate Change on Landscape Connectivity and Genetic Clusters in a Small Subtropical and Warm-Temperate Tree
Source: Front Plant Sci. 2021 Nov 10;12:671336. doi: 10.3389/fpls.2021.671336 (PMC8631755; doi:10.3389/fpls.2021.671336)
Supplement: Supplementary file 1 [file Data_Sheet_1.doc]

**Supporting information 1-Table**

**Table S1** Contemporary occurrence records (129) of *Cornus kousa* subsp. *chinensis* represented by the centroid of grid cells across the central and eastern China used in the species distribution modelling**.** Latitude and longitude in decimal degrees.

| Longitude | Latitude | Longitude | Latitude | Longitude | Latitude | Longitude | Latitude | Longitude | Latitude |
| --- | --- | --- | --- | --- | --- | --- | --- | --- | --- |
| 104.47 | 25.70 | 113.81 | 29.24 | 110.87 | 30.22 | 110.50 | 31.50 | 116.17 | 33.27 |
| 108.17 | 26.35 | 114.96 | 29.33 | 118.14 | 30.27 | 115.88 | 31.68 | 111.48 | 33.28 |
| 110.50 | 26.42 | 102.68 | 29.35 | 120.17 | 30.27 | 115.50 | 31.70 | 108.30 | 33.30 |
| 110.86 | 26.43 | 110.16 | 29.40 | 108.94 | 30.29 | 111.16 | 31.73 | 106.16 | 33.33 |
| 111.94 | 26.77 | 103.40 | 29.50 | 109.48 | 30.30 | 114.02 | 31.77 | 105.61 | 33.33 |
| 104.20 | 26.91 | 115.98 | 29.53 | 119.45 | 30.31 | 108.63 | 31.82 | 109.15 | 33.42 |
| 115.89 | 27.01 | 117.67 | 29.55 | 110.85 | 30.47 | 109.53 | 31.88 | 111.90 | 33.51 |
| 104.62 | 27.12 | 118.88 | 29.58 | 109.73 | 30.60 | 104.44 | 31.89 | 107.99 | 33.52 |
| 112.81 | 27.22 | 114.49 | 29.60 | 117.49 | 30.61 | 107.24 | 31.91 | 106.92 | 33.62 |
| 112.69 | 27.29 | 115.99 | 29.67 | 119.57 | 30.62 | 119.17 | 31.92 | 111.33 | 33.68 |
| 114.53 | 27.40 | 111.02 | 29.70 | 117.93 | 30.62 | 108.67 | 31.95 | 110.33 | 33.70 |
| 119.94 | 27.56 | 109.87 | 29.72 | 113.17 | 30.66 | 118.80 | 32.06 | 108.31 | 33.71 |
| 104.05 | 27.63 | 121.51 | 29.78 | 118.41 | 30.69 | 119.42 | 32.06 | 110.83 | 33.75 |
| 103.25 | 27.70 | 118.18 | 29.79 | 115.40 | 30.78 | 108.04 | 32.07 | 112.04 | 33.96 |
| 120.29 | 28.14 | 118.95 | 29.80 | 110.72 | 30.88 | 109.82 | 32.09 | 107.25 | 33.98 |
| 103.64 | 28.23 | 118.43 | 29.87 | 119.42 | 30.89 | 108.90 | 32.31 | 111.05 | 34.05 |
| 114.78 | 28.39 | 121.54 | 29.87 | 116.20 | 30.92 | 106.84 | 32.35 | 107.89 | 34.11 |
| 118.24 | 28.68 | 110.03 | 29.89 | 111.26 | 30.97 | 109.36 | 32.39 | 106.00 | 34.18 |
| 109.65 | 28.71 | 118.25 | 29.96 | 110.34 | 31.04 | 113.08 | 32.40 | 109.51 | 34.50 |
| 115.36 | 28.86 | 115.97 | 29.98 | 109.41 | 31.05 | 111.05 | 32.43 | 110.85 | 34.52 |
| 117.58 | 28.95 | 109.48 | 29.99 | 109.88 | 31.11 | 104.55 | 32.45 | 105.73 | 34.57 |
| 114.41 | 29.01 | 118.02 | 30.02 | 115.77 | 31.11 | 109.03 | 32.68 | 106.81 | 34.88 |
| 110.46 | 29.05 | 108.19 | 30.02 | 110.78 | 31.33 | 104.68 | 32.94 | 112.85 | 35.18 |
| 118.42 | 29.14 | 101.96 | 30.06 | 109.63 | 31.37 | 106.94 | 33.00 | 113.43 | 35.42 |
| 120.98 | 29.14 | 102.76 | 30.07 | 116.33 | 31.39 | 108.25 | 33.04 | 112.01 | 35.46 |
| 107.10 | 29.16 | 119.37 | 30.20 | 119.38 | 31.42 | 107.55 | 33.22 |  |  |

**Table S2** Sampling location of the 24 populations of *Cornus kousa* subsp. *chinensis* used for genetic analyses.

| Population location | Longitude* | Latitude* |
| --- | --- | --- |
| AF | 114.468889 | 27.275556 |
| BTM | 111.863056 | 33.500556 |
| CA | 118.951667 | 29.801389 |
| CK | 108.702222 | 32.054444 |
| DJY | 103.570556 | 31.121111 |
| EMS | 103.373611 | 29.543889 |
| HS | 112.682222 | 27.249167 |
| JGS | 114.066389 | 31.819444 |
| LD | 102.2475 | 29.893333 |
| LS | 115.987222 | 29.541111 |
| LiS | 112.040833 | 35.402778 |
| NS | 108.537222 | 33.556111 |
| TBS | 107.810833 | 33.905833 |
| TMS | 110.4675 | 29.057778 |
| TTS | 121.092222 | 29.255556 |
| TTZ | 115.771389 | 31.161667 |
| WDS | 111.050556 | 32.438889 |
| XE | 109.508611 | 29.976111 |
| XJZ | 115.998333 | 27.222778 |
| XTM | 119.500556 | 30.343611 |
| YL | 117.666111 | 29.549167 |
| ZP | 109.366667 | 31.983333 |
| LJS | 104.01 | 28.819 |
| WWS | 104.9856 | 28.322 |

* Decimal degrees

**Table S3 GenBank accession numbers of the cpDNA sequences of *Cornus kousa* subsp. *chinensis* used in the this study**

| **Taxon** | **Haplotypes** | ***rpl*14*-rpl*36** | ***trn*L-*trn*F** |
| --- | --- | --- | --- |
|  | H1 | MT796677 | MT796672 |
|  | H2 | MT796677 | MT796673 |
|  | H3 | MT796678 | MT796672 |
|  | H4 | MT796679 | MT796672 |
|  | H5 | MT796679 | MT796674 |
|  | H6 | MT796677 | MT796675 |
|  | H7 | MT796680 | MT796676 |

**Table S4** AUC and TSS values used to SDMs of *C. kousa* subsp. *chinensis*

|  | MAXENT | SRE | CTA | RF | MARS | FDA | GLM | GBM | GAM | ANN |
| --- | --- | --- | --- | --- | --- | --- | --- | --- | --- | --- |
| AUC | 0.851 | 0.806 | 0.827 | 0.919 | 0.916 | 0.920 | 0.928 | 0.931 | 0.895 | 0.860 |
| TSS | 0.658 | 0.611 | 0.648 | 0.738 | 0.722 | 0.753 | 0.762 | 0.758 | 0.698 | 0.637 |

**Table S5** The importance of environmental variables affecting the SDMs

| Variables | MAXENT | SRE | CTA | RF | MARS | FDA | GLM | GBM | GAM | ANN | Mean | SD |
| --- | --- | --- | --- | --- | --- | --- | --- | --- | --- | --- | --- | --- |
| BIO1 | 0.414 | 0.351 | 0.845 | 0.48 | 0.777 | 0.964 | 0.652 | 0.535 | 0.497 | 0.409 | 0.592 | 0.197 |
| BIO2 | 0.129 | 0.323 | 0 | 0.154 | 0.172 | 0.112 | 0.139 | 0.084 | 0.442 | 0.358 | 0.191 | 0.131 |
| BIO3 | 0.057 | 0.217 | 0 | 0.094 | 0.371 | 0.192 | 0.231 | 0.095 | 0.611 | 0.367 | 0.224 | 0.166 |
| BIO12 | 0.295 | 0.421 | 0.311 | 0.202 | 0.243 | 0.254 | 0.388 | 0.26 | 0.22 | 0.423 | 0.302 | 0.078 |
| BIO14 | 0.239 | 0.291 | 0 | 0.051 | 0.798 | 0.637 | 0.796 | 0.055 | 0.585 | 0.734 | 0.419 | 0.309 |
| BIO15 | 0.024 | 0.267 | 0 | 0.077 | 0.307 | 0.332 | 0.301 | 0.026 | 0.295 | 0.201 | 0.183 | 0.129 |
| Land cover | 0.129 | 0.019 | 0 | 0.06 | 0.057 | 0.064 | 0.055 | 0.020 | 0.183 | 0.038 | 0.063 | 0.050 |
| Elevation | 0.342 | 0.199 | 0 | 0.069 | 0 | 0 | 0.098 | 0.083 | 0.192 | 0.673 | 0.166 | 0.198 |
| Vegetation | 0.007 | 0.054 | 0 | 0.028 | 0.08 | 0.043 | 0.035 | 0.014 | 0.04 | 0.054 | 0.036 | 0.023 |

**Table S6 Proportion on ancestry for individuals of *Cornus kousa* subsp*. chinensis*, conditioned to climatic variables, inferred by Bayesian analysis of microsatellites data for current climate conditions**

|  |  |  | **Inferred Cluster** | | |  |  |
| --- | --- | --- | --- | --- | --- | --- | --- |
| **Individual** | **01** | **02** | **03** | **04** | **05** | **06** | **07** |
| **1** | 0.01453 | 0.08622 | 0.15000 | 0.60342 | 0.02898 | 0.02726 | 0.08959 |
| **2** | 0.01474 | 0.08559 | 0.15240 | 0.59918 | 0.02964 | 0.03143 | 0.08701 |
| **3** | 0.01370 | 0.08529 | 0.15015 | 0.60369 | 0.02901 | 0.03107 | 0.08709 |
| **4** | 0.01402 | 0.08485 | 0.15038 | 0.60236 | 0.02935 | 0.03169 | 0.08736 |
| **5** | 0.01418 | 0.08447 | 0.14962 | 0.60358 | 0.03040 | 0.02900 | 0.08876 |
| **6** | 0.01361 | 0.08524 | 0.15231 | 0.60167 | 0.03061 | 0.02882 | 0.08774 |
| **7** | 0.00547 | 0.00495 | 0.01340 | 0.93626 | 0.02790 | 0.00353 | 0.00849 |
| **8** | 0.00554 | 0.00532 | 0.01376 | 0.93576 | 0.02863 | 0.00333 | 0.00767 |
| **9** | 0.00523 | 0.00504 | 0.01452 | 0.93574 | 0.02842 | 0.00324 | 0.00781 |
| **10** | 0.00536 | 0.00525 | 0.01347 | 0.93721 | 0.02757 | 0.00330 | 0.00784 |
| **11** | 0.00487 | 0.00509 | 0.01450 | 0.93617 | 0.02723 | 0.00347 | 0.00868 |
| **12** | 0.00495 | 0.00482 | 0.01388 | 0.93732 | 0.02725 | 0.00345 | 0.00833 |
| **13** | 0.00515 | 0.00506 | 0.01429 | 0.93504 | 0.02809 | 0.00355 | 0.00882 |
| **14** | 0.00543 | 0.00567 | 0.01400 | 0.93630 | 0.02684 | 0.00327 | 0.00849 |
| **15** | 0.00552 | 0.00491 | 0.01381 | 0.93647 | 0.02748 | 0.00364 | 0.00818 |
| **16** | 0.00531 | 0.00532 | 0.01393 | 0.93766 | 0.02668 | 0.00303 | 0.00807 |
| **17** | 0.00537 | 0.00467 | 0.01445 | 0.93705 | 0.02722 | 0.00333 | 0.00791 |
| **18** | 0.00524 | 0.00507 | 0.01376 | 0.93638 | 0.02835 | 0.00321 | 0.00800 |
| **19** | 0.00542 | 0.00560 | 0.01410 | 0.93576 | 0.02722 | 0.00344 | 0.00847 |
| **20** | 0.00569 | 0.00465 | 0.01362 | 0.93622 | 0.02802 | 0.00332 | 0.00848 |
| **21** | 0.00194 | 0.00481 | 0.03894 | 0.93611 | 0.00047 | 0.00215 | 0.01558 |
| **22** | 0.00181 | 0.00473 | 0.03908 | 0.93619 | 0.00050 | 0.00214 | 0.01555 |
| **23** | 0.00181 | 0.00473 | 0.03998 | 0.93503 | 0.00052 | 0.00192 | 0.01602 |
| **24** | 0.01140 | 0.00616 | 0.01231 | 0.89696 | 0.02371 | 0.03818 | 0.01129 |
| **25** | 0.01048 | 0.00705 | 0.01263 | 0.89523 | 0.02395 | 0.03871 | 0.01194 |
| **26** | 0.01044 | 0.00703 | 0.01177 | 0.89603 | 0.02544 | 0.03730 | 0.01198 |
| **27** | 0.01069 | 0.00642 | 0.01240 | 0.89699 | 0.02368 | 0.03819 | 0.01164 |
| **28** | 0.01093 | 0.00621 | 0.01223 | 0.89593 | 0.02493 | 0.03795 | 0.01182 |
| **29** | 0.01501 | 0.01622 | 0.01083 | 0.16056 | 0.74547 | 0.03805 | 0.01385 |
| **30** | 0.01427 | 0.01664 | 0.01122 | 0.16297 | 0.74356 | 0.03779 | 0.01354 |
| **31** | 0.01463 | 0.01556 | 0.01035 | 0.16157 | 0.74512 | 0.03876 | 0.01401 |
| **32** | 0.01514 | 0.01774 | 0.01059 | 0.16240 | 0.74040 | 0.03819 | 0.01553 |
| **33** | 0.01449 | 0.01612 | 0.01068 | 0.16053 | 0.74536 | 0.03898 | 0.01383 |
| **34** | 0.01433 | 0.01667 | 0.01003 | 0.16177 | 0.74371 | 0.03936 | 0.01413 |
| **35** | 0.01606 | 0.01648 | 0.01019 | 0.16112 | 0.74288 | 0.03900 | 0.01427 |
| **36** | 0.02941 | 0.02856 | 0.02189 | 0.12768 | 0.41932 | 0.34808 | 0.02505 |
| **37** | 0.02925 | 0.02865 | 0.02067 | 0.12416 | 0.41938 | 0.35331 | 0.02458 |
| **38** | 0.02976 | 0.02715 | 0.02022 | 0.12663 | 0.41911 | 0.35179 | 0.02534 |
| **39** | 0.02868 | 0.02973 | 0.01977 | 0.12884 | 0.41254 | 0.35431 | 0.02611 |
| **40** | 0.02867 | 0.02907 | 0.01929 | 0.12721 | 0.41871 | 0.35173 | 0.02532 |
| **41** | 0.03052 | 0.02801 | 0.01839 | 0.12636 | 0.41646 | 0.35358 | 0.02669 |
| **42** | 0.03064 | 0.02937 | 0.02062 | 0.12570 | 0.41448 | 0.35186 | 0.02733 |
| **43** | 0.02700 | 0.03139 | 0.02002 | 0.12790 | 0.41454 | 0.35151 | 0.02763 |
| **44** | 0.02965 | 0.03031 | 0.02068 | 0.12790 | 0.42024 | 0.34489 | 0.02633 |
| **45** | 0.02840 | 0.03017 | 0.02041 | 0.13123 | 0.41997 | 0.34472 | 0.02510 |
| **46** | 0.02978 | 0.02965 | 0.01883 | 0.12992 | 0.41602 | 0.35045 | 0.02534 |
| **47** | 0.02956 | 0.02899 | 0.02046 | 0.12470 | 0.41780 | 0.35204 | 0.02645 |
| **48** | 0.02865 | 0.02970 | 0.01906 | 0.13074 | 0.41509 | 0.35046 | 0.02630 |
| **49** | 0.03227 | 0.03031 | 0.01882 | 0.12407 | 0.41844 | 0.34931 | 0.02678 |
| **50** | 0.02981 | 0.02986 | 0.02006 | 0.12616 | 0.41931 | 0.35103 | 0.02377 |
| **51** | 0.01046 | 0.03834 | 0.03125 | 0.85582 | 0.03152 | 0.01067 | 0.02194 |
| **52** | 0.01104 | 0.03769 | 0.03003 | 0.85763 | 0.03073 | 0.01073 | 0.02214 |
| **53** | 0.01025 | 0.03575 | 0.03035 | 0.85913 | 0.03153 | 0.00960 | 0.02337 |
| **54** | 0.01078 | 0.03745 | 0.03081 | 0.85787 | 0.03110 | 0.00949 | 0.02249 |
| **55** | 0.01057 | 0.03826 | 0.03071 | 0.85531 | 0.03144 | 0.01041 | 0.02330 |
| **56** | 0.01055 | 0.03788 | 0.03040 | 0.85641 | 0.03168 | 0.01033 | 0.02276 |
| **57** | 0.01072 | 0.03776 | 0.03005 | 0.85858 | 0.03054 | 0.00990 | 0.02244 |
| **58** | 0.01044 | 0.03731 | 0.03127 | 0.85687 | 0.03173 | 0.00970 | 0.02268 |
| **59** | 0.01057 | 0.03664 | 0.03003 | 0.85675 | 0.03298 | 0.01053 | 0.02251 |
| **60** | 0.01056 | 0.03710 | 0.03007 | 0.85919 | 0.03235 | 0.00924 | 0.02150 |
| **61** | 0.00347 | 0.00291 | 0.01279 | 0.96425 | 0.00127 | 0.00999 | 0.00532 |
| **62** | 0.00381 | 0.00231 | 0.01314 | 0.96453 | 0.00140 | 0.01034 | 0.00447 |
| **63** | 0.00323 | 0.00226 | 0.01330 | 0.96498 | 0.00120 | 0.01029 | 0.00474 |
| **64** | 0.00356 | 0.00281 | 0.01307 | 0.96431 | 0.00138 | 0.00989 | 0.00500 |
| **65** | 0.00340 | 0.00260 | 0.01276 | 0.96450 | 0.00144 | 0.01040 | 0.00490 |
| **66** | 0.00378 | 0.00257 | 0.01336 | 0.96355 | 0.00144 | 0.00998 | 0.00532 |
| **67** | 0.00345 | 0.00268 | 0.01268 | 0.96452 | 0.00127 | 0.01000 | 0.00540 |
| **68** | 0.00387 | 0.00223 | 0.01263 | 0.96356 | 0.00138 | 0.01091 | 0.00542 |
| **69** | 0.00344 | 0.00270 | 0.01266 | 0.96451 | 0.00129 | 0.01016 | 0.00524 |
| **70** | 0.00495 | 0.01600 | 0.01927 | 0.00641 | 0.91891 | 0.00712 | 0.02735 |
| **71** | 0.00493 | 0.01420 | 0.02010 | 0.00622 | 0.92170 | 0.00644 | 0.02641 |
| **72** | 0.00569 | 0.01468 | 0.01947 | 0.00613 | 0.92178 | 0.00681 | 0.02543 |
| **73** | 0.00513 | 0.01446 | 0.01910 | 0.00665 | 0.92174 | 0.00689 | 0.02604 |
| **74** | 0.00517 | 0.01579 | 0.01915 | 0.00640 | 0.92095 | 0.00630 | 0.02625 |
| **75** | 0.00530 | 0.01578 | 0.01869 | 0.00617 | 0.92043 | 0.00681 | 0.02683 |
| **76** | 0.00533 | 0.01563 | 0.01958 | 0.00672 | 0.91946 | 0.00689 | 0.02639 |
| **77** | 0.00516 | 0.01525 | 0.01896 | 0.00671 | 0.92100 | 0.00644 | 0.02649 |
| **78** | 0.00530 | 0.01451 | 0.01930 | 0.00639 | 0.92185 | 0.00635 | 0.02631 |
| **79** | 0.00514 | 0.01557 | 0.01927 | 0.00605 | 0.92095 | 0.00606 | 0.02696 |
| **80** | 0.00067 | 0.00278 | 0.00272 | 0.99018 | 0.00263 | 0.00004 | 0.00098 |
| **81** | 0.00061 | 0.00286 | 0.00245 | 0.99047 | 0.00255 | 0.00004 | 0.00102 |
| **82** | 0.00071 | 0.00254 | 0.00281 | 0.99071 | 0.00227 | 0.00005 | 0.00090 |
| **83** | 0.00062 | 0.00293 | 0.00249 | 0.99009 | 0.00281 | 0.00007 | 0.00099 |
| **84** | 0.00061 | 0.00297 | 0.00266 | 0.99018 | 0.00260 | 0.00004 | 0.00094 |
| **85** | 0.00039 | 0.00019 | 0.00026 | 0.99648 | 0.00245 | 0.00001 | 0.00021 |
| **86** | 0.00031 | 0.00012 | 0.00026 | 0.99638 | 0.00264 | 0.00001 | 0.00028 |
| **87** | 0.00034 | 0.00018 | 0.00028 | 0.99636 | 0.00257 | 0.00002 | 0.00024 |
| **88** | 0.00043 | 0.00015 | 0.00026 | 0.99655 | 0.00240 | 0.00003 | 0.00019 |
| **89** | 0.00036 | 0.00012 | 0.00034 | 0.99652 | 0.00239 | 0.00001 | 0.00026 |
| **90** | 0.00206 | 0.00114 | 0.00129 | 0.98588 | 0.00633 | 0.00174 | 0.00156 |
| **91** | 0.00207 | 0.00121 | 0.00106 | 0.98548 | 0.00708 | 0.00158 | 0.00152 |
| **92** | 0.00185 | 0.00140 | 0.00110 | 0.98585 | 0.00650 | 0.00162 | 0.00169 |
| **93** | 0.00190 | 0.00114 | 0.00104 | 0.98598 | 0.00673 | 0.00176 | 0.00145 |
| **94** | 0.00213 | 0.00113 | 0.00116 | 0.98555 | 0.00726 | 0.00141 | 0.00136 |
| **95** | 0.00203 | 0.00126 | 0.00117 | 0.98558 | 0.00669 | 0.00176 | 0.00150 |
| **96** | 0.00206 | 0.00107 | 0.00114 | 0.98557 | 0.00703 | 0.00159 | 0.00155 |
| **97** | 0.00107 | 0.00035 | 0.00030 | 0.98740 | 0.00994 | 0.00020 | 0.00074 |
| **98** | 0.00095 | 0.00047 | 0.00039 | 0.98772 | 0.00967 | 0.00014 | 0.00066 |
| **99** | 0.00088 | 0.00034 | 0.00040 | 0.98738 | 0.01011 | 0.00016 | 0.00073 |
| **100** | 0.00089 | 0.00033 | 0.00036 | 0.98704 | 0.01052 | 0.00018 | 0.00068 |
| **101** | 0.00083 | 0.00033 | 0.00038 | 0.98738 | 0.01023 | 0.00019 | 0.00066 |
| **102** | 0.00103 | 0.00044 | 0.00038 | 0.98712 | 0.01004 | 0.00014 | 0.00085 |
| **103** | 0.00115 | 0.00038 | 0.00034 | 0.98710 | 0.01031 | 0.00016 | 0.00056 |
| **104** | 0.00095 | 0.00044 | 0.00045 | 0.98722 | 0.00997 | 0.00017 | 0.00079 |
| **105** | 0.00103 | 0.00047 | 0.00042 | 0.98726 | 0.00985 | 0.00014 | 0.00084 |
| **106** | 0.00107 | 0.00038 | 0.00045 | 0.98729 | 0.00990 | 0.00014 | 0.00078 |
| **107** | 0.00774 | 0.01570 | 0.01208 | 0.88216 | 0.06714 | 0.00558 | 0.00962 |
| **108** | 0.00740 | 0.01445 | 0.01166 | 0.88555 | 0.06443 | 0.00572 | 0.01078 |
| **109** | 0.00736 | 0.01527 | 0.01190 | 0.88614 | 0.06390 | 0.00541 | 0.01001 |
| **110** | 0.00791 | 0.01513 | 0.01172 | 0.88408 | 0.06447 | 0.00592 | 0.01077 |
| **111** | 0.00785 | 0.01469 | 0.01115 | 0.88580 | 0.06443 | 0.00600 | 0.01007 |
| **112** | 0.00762 | 0.01555 | 0.01200 | 0.88411 | 0.06537 | 0.00518 | 0.01017 |
| **113** | 0.00682 | 0.01568 | 0.01224 | 0.88508 | 0.06483 | 0.00502 | 0.01034 |
| **114** | 0.00740 | 0.01488 | 0.01190 | 0.88606 | 0.06380 | 0.00599 | 0.00998 |
| **115** | 0.00774 | 0.01469 | 0.01101 | 0.88909 | 0.06214 | 0.00584 | 0.00949 |
| **116** | 0.00750 | 0.01534 | 0.01152 | 0.88496 | 0.06590 | 0.00482 | 0.00997 |
| **117** | 0.00738 | 0.01508 | 0.01109 | 0.88715 | 0.06448 | 0.00545 | 0.00938 |
| **118** | 0.00298 | 0.00568 | 0.14027 | 0.79579 | 0.00029 | 0.00934 | 0.04565 |
| **119** | 0.00317 | 0.00632 | 0.13838 | 0.79693 | 0.00020 | 0.00921 | 0.04579 |
| **120** | 0.00284 | 0.00589 | 0.13894 | 0.79739 | 0.00025 | 0.00951 | 0.04518 |
| **121** | 0.00323 | 0.00558 | 0.13928 | 0.79628 | 0.00025 | 0.00926 | 0.04613 |
| **122** | 0.00060 | 0.00092 | 0.00286 | 0.99202 | 0.00133 | 0.00018 | 0.00208 |
| **123** | 0.00076 | 0.00086 | 0.00299 | 0.99222 | 0.00118 | 0.00014 | 0.00184 |
| **124** | 0.00063 | 0.00078 | 0.00267 | 0.99249 | 0.00126 | 0.00013 | 0.00205 |
| **125** | 0.00058 | 0.00092 | 0.00295 | 0.99238 | 0.00117 | 0.00020 | 0.00180 |
| **126** | 0.00060 | 0.00084 | 0.00310 | 0.99225 | 0.00126 | 0.00012 | 0.00183 |
| **127** | 0.00068 | 0.00078 | 0.00291 | 0.99235 | 0.00114 | 0.00017 | 0.00197 |
| **128** | 0.00068 | 0.00090 | 0.00298 | 0.99190 | 0.00139 | 0.00017 | 0.00199 |
| **129** | 0.00061 | 0.00094 | 0.00305 | 0.99227 | 0.00116 | 0.00015 | 0.00182 |
| **130** | 0.00060 | 0.00086 | 0.00307 | 0.99223 | 0.00129 | 0.00009 | 0.00186 |
| **131** | 0.00055 | 0.00096 | 0.00321 | 0.99192 | 0.00126 | 0.00013 | 0.00196 |
| **132** | 0.00076 | 0.00084 | 0.00281 | 0.99239 | 0.00113 | 0.00016 | 0.00192 |
| **133** | 0.01297 | 0.01644 | 0.03612 | 0.82453 | 0.07217 | 0.01650 | 0.02126 |
| **134** | 0.01234 | 0.01633 | 0.03564 | 0.82493 | 0.07384 | 0.01622 | 0.02070 |
| **135** | 0.01256 | 0.01650 | 0.03677 | 0.82513 | 0.07246 | 0.01619 | 0.02038 |
| **136** | 0.01338 | 0.01651 | 0.03582 | 0.82396 | 0.07343 | 0.01644 | 0.02046 |
| **137** | 0.01283 | 0.01638 | 0.03615 | 0.82284 | 0.07435 | 0.01670 | 0.02074 |
| **138** | 0.01500 | 0.01838 | 0.02188 | 0.81295 | 0.07341 | 0.04288 | 0.01551 |
| **139** | 0.01375 | 0.01942 | 0.02265 | 0.81395 | 0.07061 | 0.04295 | 0.01666 |
| **140** | 0.01483 | 0.01787 | 0.02122 | 0.81788 | 0.07029 | 0.04079 | 0.01713 |
| **141** | 0.01577 | 0.01898 | 0.02197 | 0.81345 | 0.07296 | 0.04053 | 0.01633 |
| **142** | 0.01441 | 0.01901 | 0.02248 | 0.81475 | 0.07002 | 0.04236 | 0.01697 |
| **143** | 0.01515 | 0.01794 | 0.02169 | 0.81536 | 0.07396 | 0.04098 | 0.01493 |
| **144** | 0.01505 | 0.01859 | 0.02156 | 0.81846 | 0.07039 | 0.04018 | 0.01577 |
| **145** | 0.01456 | 0.01873 | 0.02111 | 0.81599 | 0.07026 | 0.04360 | 0.01575 |
| **146** | 0.01491 | 0.01859 | 0.02113 | 0.81714 | 0.07168 | 0.04149 | 0.01506 |
| **147** | 0.01432 | 0.01792 | 0.02181 | 0.81691 | 0.07169 | 0.04070 | 0.01664 |
| **148** | 0.01467 | 0.01859 | 0.02247 | 0.81720 | 0.07021 | 0.04201 | 0.01486 |
| **149** | 0.01389 | 0.01834 | 0.02240 | 0.81661 | 0.07226 | 0.04023 | 0.01627 |
| **150** | 0.01494 | 0.01894 | 0.02282 | 0.81302 | 0.07107 | 0.04249 | 0.01673 |
| **151** | 0.01440 | 0.01902 | 0.02251 | 0.81645 | 0.06984 | 0.04093 | 0.01686 |
| **152** | 0.01446 | 0.01975 | 0.02142 | 0.81770 | 0.06880 | 0.04107 | 0.01679 |
| **153** | 0.01473 | 0.01892 | 0.02170 | 0.81527 | 0.07214 | 0.04178 | 0.01545 |
| **154** | 0.01355 | 0.04993 | 0.43375 | 0.16058 | 0.01395 | 0.12131 | 0.20693 |
| **155** | 0.01245 | 0.04775 | 0.43477 | 0.16273 | 0.01247 | 0.12174 | 0.20810 |
| **156** | 0.01477 | 0.04983 | 0.43421 | 0.15766 | 0.01226 | 0.12119 | 0.21007 |
| **157** | 0.01436 | 0.05030 | 0.43080 | 0.16235 | 0.01252 | 0.12105 | 0.20863 |
| **158** | 0.01330 | 0.04977 | 0.42898 | 0.16392 | 0.01318 | 0.12118 | 0.20967 |
| **159** | 0.01402 | 0.04985 | 0.43290 | 0.15991 | 0.01288 | 0.12156 | 0.20889 |
| **160** | 0.01509 | 0.05011 | 0.43435 | 0.15797 | 0.01143 | 0.12249 | 0.20855 |
| **161** | 0.01411 | 0.05024 | 0.43127 | 0.16195 | 0.01216 | 0.12207 | 0.20820 |
| **162** | 0.01389 | 0.05055 | 0.43023 | 0.15967 | 0.01302 | 0.12260 | 0.21003 |
| **163** | 0.01414 | 0.05097 | 0.43307 | 0.15986 | 0.01272 | 0.12119 | 0.20805 |
| **164** | 0.00148 | 0.00329 | 0.02619 | 0.95906 | 0.00021 | 0.00136 | 0.00842 |
| **165** | 0.00141 | 0.00329 | 0.02545 | 0.95962 | 0.00022 | 0.00122 | 0.00879 |
| **166** | 0.00125 | 0.00340 | 0.02577 | 0.95965 | 0.00033 | 0.00118 | 0.00842 |
| **167** | 0.00129 | 0.00332 | 0.02507 | 0.95990 | 0.00024 | 0.00127 | 0.00891 |
| **168** | 0.00137 | 0.00316 | 0.02490 | 0.96007 | 0.00026 | 0.00140 | 0.00883 |
| **169** | 0.00134 | 0.00321 | 0.02528 | 0.96056 | 0.00025 | 0.00134 | 0.00802 |
| **170** | 0.00142 | 0.00325 | 0.02585 | 0.95929 | 0.00028 | 0.00136 | 0.00855 |
| **171** | 0.00075 | 0.00567 | 0.00872 | 0.97603 | 0.00288 | 0.00009 | 0.00586 |
| **172** | 0.00073 | 0.00572 | 0.00873 | 0.97589 | 0.00288 | 0.00005 | 0.00601 |
| **173** | 0.00075 | 0.00570 | 0.00871 | 0.97601 | 0.00281 | 0.00006 | 0.00596 |
| **174** | 0.00065 | 0.00525 | 0.00866 | 0.97693 | 0.00274 | 0.00006 | 0.00572 |
| **175** | 0.00066 | 0.00587 | 0.00857 | 0.97587 | 0.00294 | 0.00003 | 0.00606 |
| **176** | 0.00067 | 0.00552 | 0.00853 | 0.97626 | 0.00278 | 0.00008 | 0.00615 |
| **177** | 0.00077 | 0.00553 | 0.00891 | 0.97589 | 0.00290 | 0.00006 | 0.00594 |
| **178** | 0.00085 | 0.00568 | 0.00906 | 0.97557 | 0.00285 | 0.00007 | 0.00592 |
| **179** | 0.00078 | 0.00558 | 0.00841 | 0.97653 | 0.00259 | 0.00009 | 0.00601 |
| **180** | 0.00083 | 0.00568 | 0.00875 | 0.97631 | 0.00267 | 0.00007 | 0.00570 |
| **181** | 0.00066 | 0.00541 | 0.00899 | 0.97617 | 0.00287 | 0.00008 | 0.00583 |
| **182** | 0.00559 | 0.00296 | 0.00542 | 0.95883 | 0.01412 | 0.00709 | 0.00598 |
| **183** | 0.00642 | 0.00314 | 0.00518 | 0.95656 | 0.01495 | 0.00811 | 0.00564 |
| **184** | 0.00572 | 0.00302 | 0.00506 | 0.95881 | 0.01433 | 0.00717 | 0.00589 |
| **185** | 0.00551 | 0.00303 | 0.00511 | 0.95828 | 0.01422 | 0.00757 | 0.00628 |
| **186** | 0.02142 | 0.02599 | 0.03182 | 0.02026 | 0.18407 | 0.68739 | 0.02905 |
| **187** | 0.02212 | 0.02392 | 0.03173 | 0.02133 | 0.18307 | 0.68700 | 0.03083 |
| **188** | 0.02180 | 0.02623 | 0.03565 | 0.02050 | 0.18612 | 0.68128 | 0.02842 |
| **189** | 0.02284 | 0.02577 | 0.03119 | 0.02005 | 0.18374 | 0.68653 | 0.02988 |
| **190** | 0.02182 | 0.02396 | 0.03311 | 0.02160 | 0.18313 | 0.68532 | 0.03107 |
| **191** | 0.02170 | 0.02439 | 0.03234 | 0.02194 | 0.18451 | 0.68389 | 0.03124 |
| **192** | 0.02249 | 0.02373 | 0.03384 | 0.02015 | 0.18856 | 0.68168 | 0.02955 |
| **193** | 0.02141 | 0.02492 | 0.03494 | 0.02270 | 0.18107 | 0.68615 | 0.02882 |
| **194** | 0.02334 | 0.02505 | 0.03199 | 0.02167 | 0.18278 | 0.68526 | 0.02991 |
| **195** | 0.02282 | 0.02586 | 0.03369 | 0.02102 | 0.18327 | 0.68265 | 0.03069 |
| **196** | 0.02075 | 0.02536 | 0.03499 | 0.02112 | 0.18407 | 0.68404 | 0.02969 |
| **197** | 0.02131 | 0.02418 | 0.03489 | 0.02091 | 0.18224 | 0.68708 | 0.02939 |
| **198** | 0.02324 | 0.02360 | 0.03348 | 0.02242 | 0.18478 | 0.68504 | 0.02743 |
| **199** | 0.02119 | 0.02497 | 0.03438 | 0.02011 | 0.18504 | 0.68607 | 0.02825 |
| **200** | 0.02188 | 0.02507 | 0.03269 | 0.02138 | 0.18291 | 0.68482 | 0.03126 |
| **201** | 0.02268 | 0.02297 | 0.03282 | 0.02167 | 0.18637 | 0.68458 | 0.02891 |
| **202** | 0.02118 | 0.02408 | 0.03203 | 0.02294 | 0.17925 | 0.69031 | 0.03022 |
| **203** | 0.02117 | 0.02170 | 0.03299 | 0.03173 | 0.07692 | 0.78874 | 0.02675 |
| **204** | 0.02117 | 0.02166 | 0.03221 | 0.03159 | 0.07867 | 0.78780 | 0.02690 |
| **205** | 0.02204 | 0.02270 | 0.03170 | 0.02894 | 0.07793 | 0.79033 | 0.02636 |
| **206** | 0.02084 | 0.02188 | 0.03203 | 0.03036 | 0.07834 | 0.79008 | 0.02647 |
| **207** | 0.02180 | 0.02306 | 0.02950 | 0.02998 | 0.07985 | 0.79176 | 0.02405 |
| **208** | 0.02139 | 0.02386 | 0.03216 | 0.02939 | 0.07860 | 0.78922 | 0.02539 |
| **209** | 0.02464 | 0.02383 | 0.03257 | 0.03236 | 0.07891 | 0.78315 | 0.02453 |
| **210** | 0.01972 | 0.02193 | 0.03167 | 0.02740 | 0.07940 | 0.79566 | 0.02423 |
| **211** | 0.02167 | 0.02134 | 0.03072 | 0.03244 | 0.07745 | 0.79104 | 0.02534 |
| **212** | 0.02218 | 0.02081 | 0.03271 | 0.02878 | 0.07789 | 0.79302 | 0.02461 |
| **213** | 0.02239 | 0.02131 | 0.03127 | 0.03179 | 0.08181 | 0.78527 | 0.02616 |
| **214** | 0.02330 | 0.02310 | 0.03124 | 0.03092 | 0.07405 | 0.79169 | 0.02570 |
| **215** | 0.02257 | 0.02306 | 0.03351 | 0.02997 | 0.07702 | 0.79041 | 0.02347 |

**Table S7** Proportion on ancestry for individuals of *Cornus kousa* subsp*. chinensis*, conditioned to climatic variables, inferred by Bayesian analysis of microsatellites data for RCP45 climatic conditions.

|  |  |  | **Inferred Cluster** | | |  |  |
| --- | --- | --- | --- | --- | --- | --- | --- |
| **Individual** | **01** | **02** | **03** | **04** | **05** | **06** | **07** |
| **1** | 0.00000 | 0.00926 | 0.11968 | 0.00000 | 0.00000 | 0.87029 | 0.00077 |
| **2** | 0.08755 | 0.00000 | 0.01867 | 0.01042 | 0.00000 | 0.88336 | 0.00000 |
| **3** | 0.00000 | 0.00000 | 0.08596 | 0.00000 | 0.00000 | 0.91404 | 0.00000 |
| **4** | 0.00000 | 0.00000 | 0.00828 | 0.00242 | 0.00000 | 0.98930 | 0.00000 |
| **5** | 0.00000 | 0.00000 | 0.57281 | 0.00000 | 0.00000 | 0.42718 | 0.00000 |
| **6** | 0.00000 | 0.00000 | 0.00045 | 0.00011 | 0.00000 | 0.99944 | 0.00000 |
| **7** | 0.00000 | 0.00277 | 0.00094 | 0.91915 | 0.07715 | 0.00000 | 0.00000 |
| **8** | 0.00000 | 0.00002 | 0.00000 | 0.92161 | 0.07837 | 0.00000 | 0.00000 |
| **9** | 0.00000 | 0.00000 | 0.00000 | 0.99915 | 0.00085 | 0.00000 | 0.00000 |
| **10** | 0.00000 | 0.00000 | 0.00000 | 1.00000 | 0.00000 | 0.00000 | 0.00000 |
| **11** | 0.00000 | 0.00000 | 0.00000 | 0.77824 | 0.22176 | 0.00000 | 0.00000 |
| **12** | 0.00058 | 0.00000 | 0.00000 | 0.99869 | 0.00017 | 0.00055 | 0.00000 |
| **13** | 0.00000 | 0.00000 | 0.00000 | 0.99481 | 0.00497 | 0.00000 | 0.00022 |
| **14** | 0.00000 | 0.00033 | 0.00003 | 0.96899 | 0.03065 | 0.00000 | 0.00000 |
| **15** | 0.00000 | 0.00000 | 0.00026 | 0.97493 | 0.02481 | 0.00000 | 0.00000 |
| **16** | 0.00000 | 0.00003 | 0.00000 | 0.94662 | 0.05335 | 0.00000 | 0.00000 |
| **17** | 0.00000 | 0.00000 | 0.00000 | 0.99963 | 0.00037 | 0.00000 | 0.00000 |
| **18** | 0.00000 | 0.00000 | 0.00000 | 0.91774 | 0.08226 | 0.00000 | 0.00000 |
| **19** | 0.00000 | 0.00007 | 0.02739 | 0.87430 | 0.09824 | 0.00000 | 0.00000 |
| **20** | 0.00000 | 0.00000 | 0.00000 | 0.82887 | 0.17112 | 0.00000 | 0.00000 |
| **21** | 0.00000 | 0.00000 | 0.18058 | 0.67258 | 0.00000 | 0.11071 | 0.03613 |
| **22** | 0.00000 | 0.01245 | 0.02182 | 0.86718 | 0.00000 | 0.09855 | 0.00000 |
| **23** | 0.00000 | 0.00000 | 0.22428 | 0.76297 | 0.00000 | 0.01271 | 0.00004 |
| **24** | 0.00000 | 0.00001 | 0.00001 | 0.99906 | 0.00053 | 0.00040 | 0.00000 |
| **25** | 0.00000 | 0.00175 | 0.00000 | 0.99361 | 0.00394 | 0.00069 | 0.00000 |
| **26** | 0.00000 | 0.00000 | 0.00000 | 0.99243 | 0.00007 | 0.00750 | 0.00000 |
| **27** | 0.00000 | 0.00000 | 0.00000 | 0.99809 | 0.00187 | 0.00004 | 0.00000 |
| **28** | 0.00023 | 0.00000 | 0.00000 | 0.94767 | 0.05210 | 0.00000 | 0.00000 |
| **29** | 0.00000 | 0.00000 | 0.00000 | 0.00000 | 1.00000 | 0.00000 | 0.00000 |
| **30** | 0.00000 | 0.00000 | 0.00000 | 0.00003 | 0.99559 | 0.00437 | 0.00000 |
| **31** | 0.00000 | 0.00000 | 0.00000 | 0.00036 | 0.99964 | 0.00000 | 0.00000 |
| **32** | 0.11924 | 0.00000 | 0.00398 | 0.01682 | 0.85996 | 0.00000 | 0.00000 |
| **33** | 0.00000 | 0.00000 | 0.00000 | 0.00000 | 1.00000 | 0.00000 | 0.00000 |
| **34** | 0.00000 | 0.00000 | 0.00000 | 0.00011 | 0.99989 | 0.00000 | 0.00000 |
| **35** | 0.00000 | 0.00000 | 0.00000 | 0.02025 | 0.97973 | 0.00002 | 0.00000 |
| **36** | 0.00000 | 0.00000 | 0.00000 | 0.00000 | 0.89440 | 0.10560 | 0.00000 |
| **37** | 0.00000 | 0.00000 | 0.00000 | 0.00000 | 0.99767 | 0.00233 | 0.00000 |
| **38** | 0.00000 | 0.00000 | 0.00000 | 0.00321 | 0.99679 | 0.00000 | 0.00000 |
| **39** | 0.00000 | 0.00000 | 0.00000 | 0.00000 | 0.99605 | 0.00395 | 0.00000 |
| **40** | 0.00000 | 0.00000 | 0.00000 | 0.00000 | 0.00000 | 0.89896 | 0.10104 |
| **41** | 0.00000 | 0.00000 | 0.00000 | 0.00000 | 1.00000 | 0.00000 | 0.00000 |
| **42** | 0.00000 | 0.00000 | 0.00000 | 0.00000 | 0.00000 | 1.00000 | 0.00000 |
| **43** | 0.00000 | 0.00000 | 0.00000 | 0.00000 | 0.00006 | 0.99994 | 0.00000 |
| **44** | 0.00000 | 0.13838 | 0.00000 | 0.86161 | 0.00000 | 0.00000 | 0.00000 |
| **45** | 0.00000 | 0.00000 | 0.00000 | 0.00002 | 0.82567 | 0.17431 | 0.00000 |
| **46** | 0.00000 | 0.00000 | 0.00000 | 0.00000 | 0.00000 | 1.00000 | 0.00000 |
| **47** | 0.00000 | 0.00000 | 0.00000 | 0.00000 | 0.96536 | 0.03464 | 0.00000 |
| **48** | 0.00000 | 0.00104 | 0.00000 | 0.00000 | 0.00128 | 0.99768 | 0.00000 |
| **49** | 0.00006 | 0.00000 | 0.00000 | 0.00000 | 0.00676 | 0.99318 | 0.00000 |
| **50** | 0.00000 | 0.00000 | 0.00000 | 0.00081 | 0.00000 | 0.99919 | 0.00000 |
| **51** | 0.00000 | 0.33075 | 0.00000 | 0.00000 | 0.00000 | 0.66925 | 0.00000 |
| **52** | 0.00000 | 0.00000 | 0.91972 | 0.00000 | 0.00000 | 0.08028 | 0.00000 |
| **53** | 0.00000 | 0.00000 | 0.37529 | 0.01272 | 0.00000 | 0.61199 | 0.00000 |
| **54** | 0.00000 | 0.00000 | 0.00380 | 0.00000 | 0.00000 | 0.99620 | 0.00000 |
| **55** | 0.00000 | 0.00000 | 0.00000 | 0.00943 | 0.00000 | 0.99057 | 0.00000 |
| **56** | 0.00000 | 0.00000 | 0.40360 | 0.00000 | 0.00000 | 0.59640 | 0.00000 |
| **57** | 0.00000 | 0.00000 | 0.29593 | 0.00477 | 0.00000 | 0.69930 | 0.00000 |
| **58** | 0.00000 | 0.00000 | 0.00839 | 0.00055 | 0.00000 | 0.99106 | 0.00000 |
| **59** | 0.00000 | 0.00000 | 0.00690 | 0.00195 | 0.00000 | 0.99114 | 0.00001 |
| **60** | 0.00000 | 0.00000 | 0.00000 | 0.00081 | 0.00000 | 0.99876 | 0.00043 |
| **61** | 0.00000 | 0.00002 | 0.00391 | 0.99600 | 0.00000 | 0.00007 | 0.00000 |
| **62** | 0.00056 | 0.00000 | 0.00058 | 0.99617 | 0.00000 | 0.00268 | 0.00000 |
| **63** | 0.00000 | 0.00000 | 0.00046 | 0.99954 | 0.00000 | 0.00000 | 0.00000 |
| **64** | 0.00020 | 0.00000 | 0.11801 | 0.87013 | 0.00000 | 0.01166 | 0.00000 |
| **65** | 0.00000 | 0.00000 | 0.00000 | 0.92695 | 0.00000 | 0.07305 | 0.00000 |
| **66** | 0.00000 | 0.00000 | 0.00879 | 0.98723 | 0.00000 | 0.00363 | 0.00035 |
| **67** | 0.00000 | 0.00000 | 0.00450 | 0.93653 | 0.00000 | 0.05897 | 0.00000 |
| **68** | 0.00000 | 0.00000 | 0.00653 | 0.99347 | 0.00000 | 0.00000 | 0.00000 |
| **69** | 0.00000 | 0.00003 | 0.05814 | 0.94183 | 0.00000 | 0.00000 | 0.00000 |
| **70** | 0.00000 | 0.00000 | 0.00000 | 0.00000 | 0.99861 | 0.00000 | 0.00138 |
| **71** | 0.00000 | 0.00505 | 0.00001 | 0.00000 | 0.99492 | 0.00000 | 0.00002 |
| **72** | 0.00139 | 0.00000 | 0.00000 | 0.00000 | 0.99856 | 0.00000 | 0.00005 |
| **73** | 0.00000 | 0.00000 | 0.00017 | 0.00033 | 0.98445 | 0.00000 | 0.01504 |
| **74** | 0.00000 | 0.00000 | 0.00000 | 0.00000 | 0.99996 | 0.00004 | 0.00000 |
| **75** | 0.00000 | 0.00000 | 0.00000 | 0.00094 | 0.99792 | 0.00000 | 0.00114 |
| **76** | 0.00000 | 0.00789 | 0.00146 | 0.00000 | 0.98646 | 0.00000 | 0.00419 |
| **77** | 0.00000 | 0.00000 | 0.00000 | 0.00000 | 0.99992 | 0.00000 | 0.00007 |
| **78** | 0.00000 | 0.00000 | 0.00398 | 0.00010 | 0.97841 | 0.00000 | 0.01751 |
| **79** | 0.00000 | 0.00606 | 0.00065 | 0.00202 | 0.99127 | 0.00000 | 0.00000 |
| **80** | 0.00000 | 0.00000 | 0.00000 | 1.00000 | 0.00000 | 0.00000 | 0.00000 |
| **81** | 0.00000 | 0.00000 | 0.01607 | 0.98370 | 0.00000 | 0.00023 | 0.00000 |
| **82** | 0.00000 | 0.00000 | 0.03950 | 0.96046 | 0.00000 | 0.00003 | 0.00000 |
| **83** | 0.00000 | 0.00000 | 0.00066 | 0.99934 | 0.00000 | 0.00000 | 0.00000 |
| **84** | 0.00000 | 0.00000 | 0.00000 | 1.00000 | 0.00000 | 0.00000 | 0.00000 |
| **85** | 0.00000 | 0.00000 | 0.00000 | 0.99927 | 0.00073 | 0.00000 | 0.00000 |
| **86** | 0.00000 | 0.00004 | 0.00001 | 0.99957 | 0.00038 | 0.00000 | 0.00001 |
| **87** | 0.00000 | 0.00009 | 0.00043 | 0.99402 | 0.00546 | 0.00000 | 0.00000 |
| **88** | 0.00000 | 0.00000 | 0.00000 | 0.99958 | 0.00042 | 0.00000 | 0.00000 |
| **89** | 0.00000 | 0.00000 | 0.00000 | 0.99981 | 0.00018 | 0.00000 | 0.00000 |
| **90** | 0.00000 | 0.00000 | 0.00000 | 0.99991 | 0.00009 | 0.00000 | 0.00000 |
| **91** | 0.00000 | 0.00000 | 0.00000 | 0.99879 | 0.00121 | 0.00000 | 0.00000 |
| **92** | 0.00430 | 0.00000 | 0.00000 | 0.99569 | 0.00000 | 0.00000 | 0.00000 |
| **93** | 0.00004 | 0.00017 | 0.00000 | 0.99979 | 0.00000 | 0.00000 | 0.00000 |
| **94** | 0.00000 | 0.00128 | 0.00001 | 0.99796 | 0.00075 | 0.00000 | 0.00000 |
| **95** | 0.00000 | 0.00000 | 0.00000 | 0.99984 | 0.00001 | 0.00000 | 0.00015 |
| **96** | 0.00000 | 0.00038 | 0.00000 | 0.99945 | 0.00002 | 0.00015 | 0.00000 |
| **97** | 0.00000 | 0.00000 | 0.00000 | 0.99964 | 0.00036 | 0.00000 | 0.00000 |
| **98** | 0.00000 | 0.00000 | 0.00000 | 0.99817 | 0.00183 | 0.00000 | 0.00000 |
| **99** | 0.00000 | 0.00000 | 0.00000 | 1.00000 | 0.00000 | 0.00000 | 0.00000 |
| **100** | 0.00000 | 0.00000 | 0.00000 | 0.99790 | 0.00210 | 0.00000 | 0.00000 |
| **101** | 0.00000 | 0.00001 | 0.00002 | 0.99861 | 0.00137 | 0.00000 | 0.00000 |
| **102** | 0.00000 | 0.00000 | 0.00000 | 0.99459 | 0.00540 | 0.00000 | 0.00000 |
| **103** | 0.00000 | 0.00000 | 0.00000 | 1.00000 | 0.00000 | 0.00000 | 0.00000 |
| **104** | 0.00000 | 0.00000 | 0.00000 | 0.99621 | 0.00379 | 0.00000 | 0.00000 |
| **105** | 0.00000 | 0.00000 | 0.00000 | 0.99880 | 0.00120 | 0.00000 | 0.00000 |
| **106** | 0.00000 | 0.00000 | 0.00000 | 0.99527 | 0.00464 | 0.00009 | 0.00000 |
| **107** | 0.00000 | 0.00000 | 0.00000 | 0.99995 | 0.00000 | 0.00005 | 0.00000 |
| **108** | 0.00000 | 0.00011 | 0.04374 | 0.84256 | 0.11359 | 0.00000 | 0.00000 |
| **109** | 0.00000 | 0.00000 | 0.00196 | 0.99804 | 0.00000 | 0.00000 | 0.00000 |
| **110** | 0.00000 | 0.00000 | 0.00000 | 0.76219 | 0.00000 | 0.00026 | 0.23754 |
| **111** | 0.00000 | 0.00000 | 0.03739 | 0.95929 | 0.00332 | 0.00000 | 0.00000 |
| **112** | 0.00001 | 0.04332 | 0.00000 | 0.95667 | 0.00000 | 0.00000 | 0.00000 |
| **113** | 0.00000 | 0.00084 | 0.00001 | 0.93325 | 0.06590 | 0.00000 | 0.00000 |
| **114** | 0.00000 | 0.00000 | 0.00000 | 0.99984 | 0.00000 | 0.00016 | 0.00000 |
| **115** | 0.00000 | 0.00000 | 0.00000 | 0.99903 | 0.00000 | 0.00097 | 0.00000 |
| **116** | 0.54577 | 0.00000 | 0.00000 | 0.34469 | 0.10954 | 0.00000 | 0.00000 |
| **117** | 0.00000 | 0.00000 | 0.00000 | 0.98779 | 0.01221 | 0.00000 | 0.00000 |
| **118** | 0.14497 | 0.00000 | 0.30944 | 0.51561 | 0.00000 | 0.02998 | 0.00000 |
| **119** | 0.00526 | 0.00000 | 0.28871 | 0.69847 | 0.00000 | 0.00362 | 0.00394 |
| **120** | 0.00000 | 0.00000 | 0.10439 | 0.85487 | 0.00000 | 0.03965 | 0.00109 |
| **121** | 0.13811 | 0.00000 | 0.20689 | 0.61757 | 0.00000 | 0.02309 | 0.01434 |
| **122** | 0.00000 | 0.00000 | 0.00178 | 0.99752 | 0.00063 | 0.00000 | 0.00007 |
| **123** | 0.00000 | 0.00000 | 0.00701 | 0.99299 | 0.00000 | 0.00000 | 0.00000 |
| **124** | 0.00000 | 0.00138 | 0.00803 | 0.98610 | 0.00000 | 0.00000 | 0.00449 |
| **125** | 0.00000 | 0.00000 | 0.00025 | 0.99974 | 0.00000 | 0.00000 | 0.00001 |
| **126** | 0.00000 | 0.00008 | 0.00000 | 0.99991 | 0.00000 | 0.00001 | 0.00000 |
| **127** | 0.00004 | 0.00000 | 0.00000 | 0.99538 | 0.00000 | 0.00412 | 0.00046 |
| **128** | 0.00126 | 0.00000 | 0.00175 | 0.99698 | 0.00000 | 0.00000 | 0.00000 |
| **129** | 0.00000 | 0.00000 | 0.01953 | 0.97855 | 0.00000 | 0.00000 | 0.00191 |
| **130** | 0.00000 | 0.00000 | 0.02248 | 0.97547 | 0.00000 | 0.00076 | 0.00129 |
| **131** | 0.00001 | 0.00000 | 0.02997 | 0.96986 | 0.00000 | 0.00000 | 0.00016 |
| **132** | 0.00000 | 0.00669 | 0.00000 | 0.99329 | 0.00000 | 0.00000 | 0.00002 |
| **133** | 0.00000 | 0.00000 | 0.00000 | 0.91440 | 0.00000 | 0.00000 | 0.08559 |
| **134** | 0.00000 | 0.00000 | 0.00000 | 0.89363 | 0.10637 | 0.00000 | 0.00000 |
| **135** | 0.00000 | 0.03155 | 0.00000 | 0.96402 | 0.00000 | 0.00443 | 0.00000 |
| **136** | 0.00000 | 0.00000 | 0.00000 | 0.88431 | 0.11569 | 0.00000 | 0.00000 |
| **137** | 0.00000 | 0.00000 | 0.00000 | 0.99759 | 0.00241 | 0.00000 | 0.00000 |
| **138** | 0.00000 | 0.00000 | 0.00000 | 0.99972 | 0.00000 | 0.00028 | 0.00000 |
| **139** | 0.00000 | 0.00000 | 0.00000 | 0.03481 | 0.00000 | 0.96519 | 0.00000 |
| **140** | 0.09262 | 0.00000 | 0.00000 | 0.82630 | 0.00000 | 0.08108 | 0.00000 |
| **141** | 0.00000 | 0.00000 | 0.00000 | 0.96486 | 0.00000 | 0.00339 | 0.03175 |
| **142** | 0.00000 | 0.00000 | 0.00000 | 0.84258 | 0.00000 | 0.15742 | 0.00000 |
| **143** | 0.00000 | 0.38394 | 0.00000 | 0.48081 | 0.00000 | 0.00000 | 0.13525 |
| **144** | 0.00000 | 0.00000 | 0.00000 | 0.99989 | 0.00000 | 0.00011 | 0.00000 |
| **145** | 0.00000 | 0.00000 | 0.00000 | 0.98431 | 0.00000 | 0.00000 | 0.01569 |
| **146** | 0.00000 | 0.00000 | 0.00000 | 0.92911 | 0.00000 | 0.00000 | 0.07089 |
| **147** | 0.00000 | 0.00000 | 0.00000 | 0.63171 | 0.34556 | 0.02273 | 0.00000 |
| **148** | 0.00000 | 0.00000 | 0.00000 | 0.98554 | 0.00000 | 0.01445 | 0.00000 |
| **149** | 0.54889 | 0.00000 | 0.00000 | 0.45111 | 0.00000 | 0.00000 | 0.00000 |
| **150** | 0.00000 | 0.00000 | 0.00000 | 0.99987 | 0.00000 | 0.00013 | 0.00000 |
| **151** | 0.00000 | 0.00000 | 0.00000 | 0.99990 | 0.00000 | 0.00010 | 0.00000 |
| **152** | 0.00000 | 0.00000 | 0.00000 | 1.00000 | 0.00000 | 0.00000 | 0.00000 |
| **153** | 0.00000 | 0.13147 | 0.00000 | 0.86742 | 0.00111 | 0.00000 | 0.00000 |
| **154** | 0.00000 | 0.00000 | 0.07500 | 0.00000 | 0.00000 | 0.92500 | 0.00000 |
| **155** | 0.00000 | 0.00000 | 0.01361 | 0.00000 | 0.00000 | 0.98639 | 0.00000 |
| **156** | 0.00000 | 0.07050 | 0.30044 | 0.00000 | 0.00000 | 0.62833 | 0.00073 |
| **157** | 0.00000 | 0.00000 | 0.01003 | 0.00000 | 0.00000 | 0.95515 | 0.03482 |
| **158** | 0.00000 | 0.00000 | 0.06655 | 0.00000 | 0.00000 | 0.93345 | 0.00000 |
| **159** | 0.00639 | 0.00000 | 0.02299 | 0.00000 | 0.00000 | 0.95697 | 0.01364 |
| **160** | 0.00000 | 0.00000 | 0.15926 | 0.00000 | 0.00000 | 0.84067 | 0.00007 |
| **161** | 0.00000 | 0.00000 | 0.08702 | 0.00000 | 0.00000 | 0.91219 | 0.00080 |
| **162** | 0.00485 | 0.00890 | 0.02441 | 0.00000 | 0.00000 | 0.94870 | 0.01315 |
| **163** | 0.00000 | 0.00000 | 0.07869 | 0.00000 | 0.02469 | 0.89234 | 0.00428 |
| **164** | 0.00000 | 0.00000 | 0.12439 | 0.86845 | 0.00000 | 0.00614 | 0.00102 |
| **165** | 0.00000 | 0.00000 | 0.11250 | 0.86756 | 0.00000 | 0.01995 | 0.00000 |
| **166** | 0.00000 | 0.00000 | 0.00492 | 0.99507 | 0.00000 | 0.00001 | 0.00000 |
| **167** | 0.00000 | 0.00389 | 0.01372 | 0.86118 | 0.00000 | 0.12121 | 0.00000 |
| **168** | 0.00000 | 0.00000 | 0.00005 | 0.99215 | 0.00000 | 0.00764 | 0.00016 |
| **169** | 0.00000 | 0.00000 | 0.00025 | 0.95149 | 0.00000 | 0.04826 | 0.00000 |
| **170** | 0.00000 | 0.00000 | 0.00247 | 0.93172 | 0.00000 | 0.06581 | 0.00000 |
| **171** | 0.00000 | 0.00000 | 0.03886 | 0.94818 | 0.00000 | 0.00000 | 0.01296 |
| **172** | 0.00000 | 0.00000 | 0.00055 | 0.99945 | 0.00000 | 0.00000 | 0.00000 |
| **173** | 0.00000 | 0.00000 | 0.03136 | 0.96818 | 0.00000 | 0.00046 | 0.00000 |
| **174** | 0.00000 | 0.00000 | 0.01717 | 0.98279 | 0.00004 | 0.00000 | 0.00001 |
| **175** | 0.00001 | 0.00000 | 0.00703 | 0.99293 | 0.00000 | 0.00003 | 0.00000 |
| **176** | 0.00000 | 0.00117 | 0.00073 | 0.99379 | 0.00000 | 0.00431 | 0.00000 |
| **177** | 0.00440 | 0.00059 | 0.15763 | 0.83638 | 0.00000 | 0.00100 | 0.00000 |
| **178** | 0.00000 | 0.00000 | 0.01223 | 0.98776 | 0.00000 | 0.00000 | 0.00001 |
| **179** | 0.00001 | 0.00000 | 0.00675 | 0.99236 | 0.00000 | 0.00082 | 0.00006 |
| **180** | 0.00000 | 0.00036 | 0.00006 | 0.99954 | 0.00000 | 0.00003 | 0.00000 |
| **181** | 0.00000 | 0.00052 | 0.00033 | 0.98873 | 0.00000 | 0.00000 | 0.01042 |
| **182** | 0.00007 | 0.00000 | 0.00000 | 0.96349 | 0.03644 | 0.00001 | 0.00000 |
| **183** | 0.00000 | 0.00001 | 0.00001 | 0.96593 | 0.03355 | 0.00049 | 0.00000 |
| **184** | 0.00000 | 0.00000 | 0.00000 | 0.98972 | 0.01028 | 0.00000 | 0.00000 |
| **185** | 0.00000 | 0.00560 | 0.00000 | 0.99440 | 0.00000 | 0.00000 | 0.00000 |
| **186** | 0.00000 | 0.00000 | 0.00000 | 0.00000 | 0.00000 | 1.00000 | 0.00000 |
| **187** | 0.00000 | 0.00000 | 0.00000 | 0.00000 | 0.00000 | 1.00000 | 0.00000 |
| **188** | 0.00000 | 0.00000 | 0.00000 | 0.00000 | 0.00053 | 0.96462 | 0.03484 |
| **189** | 0.00274 | 0.00000 | 0.00000 | 0.00000 | 0.00000 | 0.99656 | 0.00070 |
| **190** | 0.00000 | 0.00000 | 0.00000 | 0.00000 | 0.00000 | 1.00000 | 0.00000 |
| **191** | 0.00000 | 0.00000 | 0.00000 | 0.00000 | 0.00000 | 1.00000 | 0.00000 |
| **192** | 0.00000 | 0.00000 | 0.00000 | 0.00000 | 0.00000 | 1.00000 | 0.00000 |
| **193** | 0.00000 | 0.00000 | 0.00000 | 0.00000 | 0.00000 | 1.00000 | 0.00000 |
| **194** | 0.00000 | 0.00000 | 0.00000 | 0.00000 | 0.00000 | 1.00000 | 0.00000 |
| **195** | 0.00000 | 0.00000 | 0.00000 | 0.00000 | 0.00000 | 1.00000 | 0.00000 |
| **196** | 0.00000 | 0.00000 | 0.00000 | 0.00000 | 0.00002 | 0.99998 | 0.00000 |
| **197** | 0.00000 | 0.00000 | 0.00000 | 0.00001 | 0.00000 | 0.99999 | 0.00000 |
| **198** | 0.00000 | 0.00000 | 0.00004 | 0.00000 | 0.00000 | 0.99996 | 0.00000 |
| **199** | 0.00000 | 0.00000 | 0.00000 | 0.00000 | 0.00000 | 1.00000 | 0.00000 |
| **200** | 0.00000 | 0.00000 | 0.00000 | 0.00000 | 0.00000 | 1.00000 | 0.00000 |
| **201** | 0.00000 | 0.00000 | 0.00000 | 0.00001 | 0.00000 | 0.99999 | 0.00000 |
| **202** | 0.00000 | 0.00000 | 0.00000 | 0.00000 | 0.00014 | 0.99986 | 0.00000 |
| **203** | 0.00000 | 0.00000 | 0.00000 | 0.00000 | 0.00000 | 1.00000 | 0.00000 |
| **204** | 0.00000 | 0.00000 | 0.00000 | 0.00000 | 0.00000 | 0.99999 | 0.00001 |
| **205** | 0.00000 | 0.00000 | 0.00000 | 0.00000 | 0.00000 | 1.00000 | 0.00000 |
| **206** | 0.00000 | 0.00000 | 0.00000 | 0.00000 | 0.00000 | 1.00000 | 0.00000 |
| **207** | 0.00000 | 0.00000 | 0.00405 | 0.00000 | 0.00000 | 0.99595 | 0.00000 |
| **208** | 0.00000 | 0.00000 | 0.00000 | 0.00000 | 0.00000 | 1.00000 | 0.00000 |
| **209** | 0.00000 | 0.00000 | 0.00000 | 0.00000 | 0.00000 | 1.00000 | 0.00000 |
| **210** | 0.00000 | 0.00000 | 0.00000 | 0.00000 | 0.00000 | 1.00000 | 0.00000 |
| **211** | 0.00000 | 0.00000 | 0.00000 | 0.00000 | 0.00000 | 1.00000 | 0.00000 |
| **212** | 0.30024 | 0.00000 | 0.00000 | 0.00000 | 0.00000 | 0.69976 | 0.00000 |
| **213** | 0.00000 | 0.00000 | 0.00000 | 0.00000 | 0.00000 | 1.00000 | 0.00000 |
| **214** | 0.00000 | 0.00000 | 0.00000 | 0.00000 | 0.00000 | 1.00000 | 0.00000 |
| **215** | 0.00407 | 0.00000 | 0.00000 | 0.00000 | 0.00000 | 0.99592 | 0.00000 |

**Table S8** Proportion on ancestry for individuals of *Cornus kousa* subsp*. chinensis*, conditioned to environmental variables, inferred by Bayesian analysis of microsatellites data for RCP85 climatic conditions.

|  |  |  | **Inferred Cluster** | | |  |  |
| --- | --- | --- | --- | --- | --- | --- | --- |
| **Individual** | **01** | **02** | **03** | **04** | **05** | **06** | **07** |
| **1** | 0.00000 | 0.00000 | 0.00405 | 0.00000 | 0.00000 | 0.99595 | 0.00000 |
| **2** | 0.00000 | 0.00000 | 0.00007 | 0.00000 | 0.00000 | 0.99993 | 0.00000 |
| **3** | 0.00000 | 0.00000 | 0.00000 | 0.00000 | 0.00000 | 1.00000 | 0.00000 |
| **4** | 0.00000 | 0.00000 | 0.00282 | 0.00000 | 0.00000 | 0.99718 | 0.00000 |
| **5** | 0.00000 | 0.00000 | 0.00142 | 0.00000 | 0.00000 | 0.99858 | 0.00000 |
| **6** | 0.00000 | 0.00000 | 0.00159 | 0.00000 | 0.00000 | 0.99841 | 0.00000 |
| **7** | 0.00000 | 0.00000 | 0.00000 | 0.99995 | 0.00000 | 0.00005 | 0.00000 |
| **8** | 0.00000 | 0.00000 | 0.00000 | 0.85133 | 0.00000 | 0.14867 | 0.00000 |
| **9** | 0.00000 | 0.00000 | 0.00000 | 0.94560 | 0.00000 | 0.00805 | 0.04635 |
| **10** | 0.00000 | 0.00000 | 0.00000 | 1.00000 | 0.00000 | 0.00000 | 0.00000 |
| **11** | 0.00000 | 0.00000 | 0.00000 | 0.99688 | 0.00311 | 0.00000 | 0.00000 |
| **12** | 0.00000 | 0.00000 | 0.00000 | 0.96160 | 0.00000 | 0.03840 | 0.00000 |
| **13** | 0.00000 | 0.00000 | 0.00000 | 0.71004 | 0.00000 | 0.28996 | 0.00000 |
| **14** | 0.00000 | 0.00000 | 0.00012 | 0.99988 | 0.00000 | 0.00000 | 0.00000 |
| **15** | 0.00000 | 0.00000 | 0.00000 | 0.99969 | 0.00031 | 0.00000 | 0.00000 |
| **16** | 0.00000 | 0.00000 | 0.00000 | 0.99038 | 0.00000 | 0.00962 | 0.00000 |
| **17** | 0.00000 | 0.00000 | 0.00000 | 0.99992 | 0.00000 | 0.00007 | 0.00000 |
| **18** | 0.00000 | 0.00000 | 0.00000 | 1.00000 | 0.00000 | 0.00000 | 0.00000 |
| **19** | 0.00000 | 0.00000 | 0.00024 | 0.99970 | 0.00000 | 0.00006 | 0.00000 |
| **20** | 0.00000 | 0.00000 | 0.00000 | 0.99732 | 0.00000 | 0.00268 | 0.00000 |
| **21** | 0.00000 | 0.00001 | 0.00616 | 0.00000 | 0.00000 | 0.99383 | 0.00000 |
| **22** | 0.00000 | 0.00000 | 0.00019 | 0.00000 | 0.00000 | 0.99801 | 0.00180 |
| **23** | 0.00000 | 0.00000 | 0.00140 | 0.00000 | 0.00000 | 0.99822 | 0.00038 |
| **24** | 0.00000 | 0.10192 | 0.00000 | 0.89808 | 0.00000 | 0.00000 | 0.00000 |
| **25** | 0.00000 | 0.00000 | 0.00000 | 0.99849 | 0.00129 | 0.00023 | 0.00000 |
| **26** | 0.00000 | 0.00000 | 0.00000 | 0.73583 | 0.00000 | 0.26417 | 0.00000 |
| **27** | 0.00000 | 0.00000 | 0.00011 | 0.99989 | 0.00000 | 0.00000 | 0.00000 |
| **28** | 0.00001 | 0.00000 | 0.00000 | 0.68582 | 0.00158 | 0.04131 | 0.27128 |
| **29** | 0.00000 | 0.00019 | 0.00000 | 0.44045 | 0.06266 | 0.00035 | 0.49635 |
| **30** | 0.00000 | 0.00000 | 0.00000 | 0.13299 | 0.86701 | 0.00000 | 0.00000 |
| **31** | 0.00000 | 0.00000 | 0.00000 | 0.03766 | 0.05543 | 0.90691 | 0.00000 |
| **32** | 0.00000 | 0.00000 | 0.00000 | 0.25540 | 0.14427 | 0.60034 | 0.00000 |
| **33** | 0.40713 | 0.00000 | 0.00000 | 0.16057 | 0.43028 | 0.00203 | 0.00000 |
| **34** | 0.00000 | 0.00000 | 0.00000 | 0.00000 | 0.94966 | 0.05034 | 0.00000 |
| **35** | 0.00000 | 0.00000 | 0.00000 | 0.00000 | 1.00000 | 0.00000 | 0.00000 |
| **36** | 0.00000 | 0.00000 | 0.00000 | 0.00006 | 0.76854 | 0.23139 | 0.00000 |
| **37** | 0.00000 | 0.00000 | 0.00000 | 0.00000 | 0.00000 | 1.00000 | 0.00000 |
| **38** | 0.00000 | 0.00000 | 0.00000 | 0.00000 | 0.00212 | 0.99788 | 0.00000 |
| **39** | 0.00000 | 0.00000 | 0.00000 | 0.00000 | 0.00001 | 0.99999 | 0.00000 |
| **40** | 0.00000 | 0.00000 | 0.00000 | 0.00000 | 0.00000 | 1.00000 | 0.00000 |
| **41** | 0.00000 | 0.00000 | 0.00001 | 0.00000 | 0.99251 | 0.00749 | 0.00000 |
| **42** | 0.00000 | 0.00000 | 0.00000 | 0.00000 | 1.00000 | 0.00000 | 0.00000 |
| **43** | 0.00000 | 0.00000 | 0.00000 | 0.00000 | 0.66673 | 0.07578 | 0.25749 |
| **44** | 0.00000 | 0.00000 | 0.00000 | 0.00000 | 0.00024 | 0.99976 | 0.00000 |
| **45** | 0.00000 | 0.00000 | 0.00000 | 0.00000 | 0.00000 | 1.00000 | 0.00000 |
| **46** | 0.00000 | 0.00000 | 0.00000 | 0.00000 | 0.00241 | 0.99759 | 0.00000 |
| **47** | 0.00000 | 0.00000 | 0.00000 | 0.00000 | 0.00000 | 1.00000 | 0.00000 |
| **48** | 0.00000 | 0.00000 | 0.00000 | 0.00000 | 0.88033 | 0.11967 | 0.00000 |
| **49** | 0.00000 | 0.00000 | 0.00000 | 0.00000 | 0.00000 | 1.00000 | 0.00000 |
| **50** | 0.00000 | 0.00006 | 0.00000 | 0.00000 | 0.00000 | 0.99994 | 0.00000 |
| **51** | 0.00000 | 0.00000 | 0.00022 | 0.00000 | 0.00000 | 0.99977 | 0.00001 |
| **52** | 0.00000 | 0.00000 | 0.00000 | 0.00000 | 0.00000 | 1.00000 | 0.00000 |
| **53** | 0.00000 | 0.00000 | 0.00000 | 0.00000 | 0.00000 | 1.00000 | 0.00000 |
| **54** | 0.00000 | 0.00000 | 0.00000 | 0.00000 | 0.00000 | 1.00000 | 0.00000 |
| **55** | 0.00000 | 0.00000 | 0.00000 | 0.00000 | 0.00000 | 1.00000 | 0.00000 |
| **56** | 0.00000 | 0.00000 | 0.00002 | 0.00000 | 0.00000 | 0.99998 | 0.00000 |
| **57** | 0.00000 | 0.00000 | 0.00006 | 0.00000 | 0.00000 | 0.99994 | 0.00000 |
| **58** | 0.00000 | 0.00000 | 0.00000 | 0.00000 | 0.00000 | 1.00000 | 0.00000 |
| **59** | 0.00000 | 0.00000 | 0.00310 | 0.00000 | 0.00000 | 0.99690 | 0.00000 |
| **60** | 0.00000 | 0.00000 | 0.00000 | 0.00000 | 0.00000 | 1.00000 | 0.00000 |
| **61** | 0.00000 | 0.00000 | 0.00078 | 0.08179 | 0.00000 | 0.91743 | 0.00000 |
| **62** | 0.00000 | 0.00000 | 0.04967 | 0.08842 | 0.00000 | 0.86192 | 0.00000 |
| **63** | 0.00000 | 0.00000 | 0.95129 | 0.03040 | 0.00000 | 0.01607 | 0.00223 |
| **64** | 0.00000 | 0.00000 | 0.00013 | 0.00471 | 0.00000 | 0.99516 | 0.00000 |
| **65** | 0.00000 | 0.00000 | 0.00189 | 0.29791 | 0.00000 | 0.70020 | 0.00000 |
| **66** | 0.00000 | 0.00000 | 0.15279 | 0.14493 | 0.00000 | 0.70226 | 0.00002 |
| **67** | 0.00000 | 0.00000 | 0.00014 | 0.55564 | 0.00000 | 0.44422 | 0.00000 |
| **68** | 0.00000 | 0.00000 | 0.02407 | 0.81945 | 0.00000 | 0.15578 | 0.00070 |
| **69** | 0.00000 | 0.00000 | 0.00000 | 0.42172 | 0.00000 | 0.57828 | 0.00000 |
| **70** | 0.00000 | 0.00000 | 0.00000 | 0.00000 | 1.00000 | 0.00000 | 0.00000 |
| **71** | 0.00000 | 0.00000 | 0.00000 | 0.00000 | 0.99980 | 0.00000 | 0.00020 |
| **72** | 0.00000 | 0.00000 | 0.00000 | 0.00000 | 0.99868 | 0.00058 | 0.00073 |
| **73** | 0.00001 | 0.00000 | 0.00116 | 0.00000 | 0.99787 | 0.00000 | 0.00096 |
| **74** | 0.00000 | 0.00000 | 0.00000 | 0.00000 | 1.00000 | 0.00000 | 0.00000 |
| **75** | 0.00000 | 0.00000 | 0.01833 | 0.00082 | 0.98085 | 0.00000 | 0.00000 |
| **76** | 0.00000 | 0.00000 | 0.00000 | 0.00000 | 1.00000 | 0.00000 | 0.00000 |
| **77** | 0.00000 | 0.00000 | 0.00000 | 0.00000 | 1.00000 | 0.00000 | 0.00000 |
| **78** | 0.00000 | 0.00000 | 0.00000 | 0.00002 | 0.99956 | 0.00000 | 0.00042 |
| **79** | 0.00000 | 0.00000 | 0.00000 | 0.00000 | 0.99962 | 0.00038 | 0.00000 |
| **80** | 0.00000 | 0.00000 | 0.00000 | 0.00000 | 0.00000 | 1.00000 | 0.00000 |
| **81** | 0.00000 | 0.00000 | 0.10372 | 0.00000 | 0.00000 | 0.89628 | 0.00000 |
| **82** | 0.00000 | 0.00000 | 0.00006 | 0.00000 | 0.00000 | 0.99994 | 0.00000 |
| **83** | 0.00000 | 0.00000 | 0.04682 | 0.00000 | 0.00000 | 0.95318 | 0.00000 |
| **84** | 0.00000 | 0.00000 | 0.00001 | 0.00000 | 0.00000 | 0.99999 | 0.00000 |
| **85** | 0.00000 | 0.00000 | 0.00000 | 0.97777 | 0.02223 | 0.00000 | 0.00000 |
| **86** | 0.00062 | 0.00000 | 0.00001 | 0.99845 | 0.00092 | 0.00000 | 0.00000 |
| **87** | 0.00004 | 0.00000 | 0.00000 | 0.99986 | 0.00010 | 0.00000 | 0.00000 |
| **88** | 0.00000 | 0.00000 | 0.00002 | 0.99979 | 0.00008 | 0.00011 | 0.00000 |
| **89** | 0.00000 | 0.00000 | 0.00024 | 0.99971 | 0.00006 | 0.00000 | 0.00000 |
| **90** | 0.00000 | 0.00000 | 0.00000 | 1.00000 | 0.00000 | 0.00000 | 0.00000 |
| **91** | 0.00000 | 0.00000 | 0.00000 | 0.99994 | 0.00002 | 0.00004 | 0.00000 |
| **92** | 0.00023 | 0.00000 | 0.00000 | 0.99977 | 0.00000 | 0.00000 | 0.00000 |
| **93** | 0.00001 | 0.00000 | 0.00000 | 0.99974 | 0.00000 | 0.00025 | 0.00000 |
| **94** | 0.00000 | 0.00000 | 0.00000 | 1.00000 | 0.00000 | 0.00000 | 0.00000 |
| **95** | 0.00002 | 0.00000 | 0.00000 | 0.99990 | 0.00000 | 0.00008 | 0.00000 |
| **96** | 0.00000 | 0.00000 | 0.00000 | 0.99663 | 0.00336 | 0.00001 | 0.00000 |
| **97** | 0.00000 | 0.00000 | 0.00000 | 1.00000 | 0.00000 | 0.00000 | 0.00000 |
| **98** | 0.00000 | 0.00000 | 0.00000 | 0.99996 | 0.00004 | 0.00000 | 0.00000 |
| **99** | 0.00015 | 0.00000 | 0.00000 | 0.99977 | 0.00000 | 0.00008 | 0.00000 |
| **100** | 0.00000 | 0.00000 | 0.00000 | 1.00000 | 0.00000 | 0.00000 | 0.00000 |
| **101** | 0.00000 | 0.00000 | 0.00000 | 0.99998 | 0.00000 | 0.00002 | 0.00000 |
| **102** | 0.00015 | 0.00002 | 0.00001 | 0.99977 | 0.00006 | 0.00000 | 0.00000 |
| **103** | 0.00000 | 0.00000 | 0.00000 | 0.99962 | 0.00038 | 0.00000 | 0.00000 |
| **104** | 0.00000 | 0.00004 | 0.00000 | 0.99996 | 0.00000 | 0.00000 | 0.00000 |
| **105** | 0.00000 | 0.00000 | 0.00000 | 1.00000 | 0.00000 | 0.00000 | 0.00000 |
| **106** | 0.00000 | 0.00000 | 0.00000 | 0.99849 | 0.00000 | 0.00151 | 0.00000 |
| **107** | 0.00000 | 0.00000 | 0.00002 | 0.00000 | 0.00000 | 0.99998 | 0.00000 |
| **108** | 0.00000 | 0.00013 | 0.00000 | 0.00999 | 0.00000 | 0.98988 | 0.00000 |
| **109** | 0.00000 | 0.00000 | 0.00001 | 0.00000 | 0.00000 | 0.99999 | 0.00000 |
| **110** | 0.00000 | 0.00208 | 0.00000 | 0.00000 | 0.00000 | 0.99792 | 0.00000 |
| **111** | 0.00000 | 0.00000 | 0.00000 | 0.00000 | 0.00000 | 1.00000 | 0.00000 |
| **112** | 0.00000 | 0.00000 | 0.00000 | 0.00000 | 0.00000 | 1.00000 | 0.00000 |
| **113** | 0.00000 | 0.00000 | 0.00000 | 0.00001 | 0.00000 | 0.99999 | 0.00000 |
| **114** | 0.00000 | 0.00000 | 0.00000 | 0.00000 | 0.00000 | 1.00000 | 0.00000 |
| **115** | 0.00000 | 0.00000 | 0.00006 | 0.00000 | 0.00000 | 0.99994 | 0.00000 |
| **116** | 0.00000 | 0.00000 | 0.00000 | 0.00000 | 0.00000 | 1.00000 | 0.00000 |
| **117** | 0.00000 | 0.00000 | 0.00000 | 0.00000 | 0.00000 | 1.00000 | 0.00000 |
| **118** | 0.00000 | 0.00000 | 0.00214 | 0.00000 | 0.00000 | 0.99785 | 0.00001 |
| **119** | 0.00000 | 0.00000 | 0.00232 | 0.00000 | 0.00000 | 0.99722 | 0.00046 |
| **120** | 0.00000 | 0.00000 | 0.00884 | 0.00000 | 0.00000 | 0.99116 | 0.00000 |
| **121** | 0.00000 | 0.00000 | 0.00949 | 0.00000 | 0.00000 | 0.99051 | 0.00000 |
| **122** | 0.00000 | 0.00000 | 0.01885 | 0.50815 | 0.00000 | 0.47298 | 0.00001 |
| **123** | 0.00000 | 0.00000 | 0.18996 | 0.35340 | 0.00000 | 0.45664 | 0.00000 |
| **124** | 0.00000 | 0.00000 | 0.00026 | 0.48007 | 0.00000 | 0.51967 | 0.00000 |
| **125** | 0.00001 | 0.00000 | 0.17944 | 0.09085 | 0.00000 | 0.72970 | 0.00000 |
| **126** | 0.00000 | 0.00000 | 0.00472 | 0.44415 | 0.00000 | 0.25253 | 0.29860 |
| **127** | 0.00000 | 0.00000 | 0.01775 | 0.05109 | 0.00000 | 0.92890 | 0.00226 |
| **128** | 0.00000 | 0.00000 | 0.02042 | 0.51253 | 0.00000 | 0.46706 | 0.00000 |
| **129** | 0.00000 | 0.00000 | 0.01970 | 0.42674 | 0.00000 | 0.55337 | 0.00019 |
| **130** | 0.00000 | 0.00000 | 0.02247 | 0.63347 | 0.00000 | 0.34324 | 0.00082 |
| **131** | 0.00000 | 0.00000 | 0.01104 | 0.70895 | 0.00000 | 0.28000 | 0.00000 |
| **132** | 0.00002 | 0.00000 | 0.06298 | 0.43971 | 0.00000 | 0.31323 | 0.18406 |
| **133** | 0.00000 | 0.00000 | 0.00000 | 1.00000 | 0.00000 | 0.00000 | 0.00000 |
| **134** | 0.00000 | 0.00000 | 0.00000 | 0.92848 | 0.00000 | 0.07152 | 0.00000 |
| **135** | 0.00000 | 0.00000 | 0.20086 | 0.79913 | 0.00000 | 0.00001 | 0.00000 |
| **136** | 0.00000 | 0.00000 | 0.00000 | 0.99204 | 0.00000 | 0.00796 | 0.00000 |
| **137** | 0.35142 | 0.00001 | 0.00000 | 0.00861 | 0.00000 | 0.63995 | 0.00000 |
| **138** | 0.00000 | 0.00000 | 0.18593 | 0.00000 | 0.00000 | 0.81407 | 0.00000 |
| **139** | 0.00000 | 0.00003 | 0.00000 | 0.00031 | 0.00000 | 0.99966 | 0.00000 |
| **140** | 0.00000 | 0.00000 | 0.00000 | 0.00000 | 0.00000 | 1.00000 | 0.00000 |
| **141** | 0.00000 | 0.00000 | 0.00000 | 0.00000 | 0.00000 | 1.00000 | 0.00000 |
| **142** | 0.00000 | 0.00000 | 0.00000 | 0.00000 | 0.00000 | 1.00000 | 0.00000 |
| **143** | 0.00000 | 0.00000 | 0.00000 | 0.04627 | 0.00000 | 0.95373 | 0.00000 |
| **144** | 0.00000 | 0.00000 | 0.00000 | 0.00000 | 0.63839 | 0.36161 | 0.00000 |
| **145** | 0.00000 | 0.00000 | 0.00113 | 0.00000 | 0.00000 | 0.99887 | 0.00000 |
| **146** | 0.00000 | 0.00000 | 0.06974 | 0.33666 | 0.00000 | 0.59360 | 0.00000 |
| **147** | 0.00000 | 0.00000 | 0.00001 | 0.40461 | 0.00000 | 0.59538 | 0.00000 |
| **148** | 0.00000 | 0.00000 | 0.00000 | 0.00000 | 0.00000 | 1.00000 | 0.00000 |
| **149** | 0.00000 | 0.00000 | 0.00000 | 0.09536 | 0.00000 | 0.90464 | 0.00000 |
| **150** | 0.00000 | 0.00000 | 0.00000 | 0.00000 | 0.00000 | 1.00000 | 0.00000 |
| **151** | 0.00000 | 0.00000 | 0.00000 | 0.00004 | 0.00000 | 0.99996 | 0.00000 |
| **152** | 0.00000 | 0.15768 | 0.00000 | 0.00000 | 0.00000 | 0.84232 | 0.00000 |
| **153** | 0.00000 | 0.00000 | 0.00000 | 0.00102 | 0.00000 | 0.99898 | 0.00000 |
| **154** | 0.00000 | 0.00000 | 0.00509 | 0.00000 | 0.00000 | 0.99491 | 0.00000 |
| **155** | 0.00000 | 0.00000 | 0.00322 | 0.00000 | 0.00000 | 0.99678 | 0.00000 |
| **156** | 0.00000 | 0.00000 | 0.00729 | 0.00000 | 0.00000 | 0.99238 | 0.00032 |
| **157** | 0.00000 | 0.00000 | 0.00212 | 0.00000 | 0.00000 | 0.99788 | 0.00000 |
| **158** | 0.00000 | 0.00000 | 0.00165 | 0.00000 | 0.00000 | 0.99834 | 0.00002 |
| **159** | 0.00000 | 0.00000 | 0.00500 | 0.00000 | 0.00000 | 0.99500 | 0.00000 |
| **160** | 0.00000 | 0.00000 | 0.00655 | 0.00000 | 0.00000 | 0.99281 | 0.00064 |
| **161** | 0.00000 | 0.00000 | 0.00291 | 0.00000 | 0.00000 | 0.99709 | 0.00000 |
| **162** | 0.00000 | 0.00000 | 0.00097 | 0.00000 | 0.00000 | 0.99810 | 0.00093 |
| **163** | 0.00000 | 0.00000 | 0.00066 | 0.00000 | 0.00000 | 0.99934 | 0.00000 |
| **164** | 0.00000 | 0.00000 | 0.00465 | 0.00000 | 0.00000 | 0.99535 | 0.00000 |
| **165** | 0.00000 | 0.00000 | 0.01773 | 0.00000 | 0.00000 | 0.98227 | 0.00000 |
| **166** | 0.00000 | 0.00000 | 0.00208 | 0.00000 | 0.00000 | 0.99792 | 0.00000 |
| **167** | 0.00000 | 0.00000 | 0.00052 | 0.00000 | 0.00000 | 0.99948 | 0.00000 |
| **168** | 0.00000 | 0.00000 | 0.00675 | 0.00000 | 0.00000 | 0.99325 | 0.00000 |
| **169** | 0.00000 | 0.00000 | 0.00603 | 0.00000 | 0.00000 | 0.99397 | 0.00000 |
| **170** | 0.00000 | 0.00000 | 0.00938 | 0.00000 | 0.00000 | 0.99062 | 0.00000 |
| **171** | 0.00000 | 0.00000 | 0.01243 | 0.00807 | 0.00000 | 0.97950 | 0.00000 |
| **172** | 0.00000 | 0.00000 | 0.07038 | 0.00000 | 0.00000 | 0.92962 | 0.00000 |
| **173** | 0.00000 | 0.00000 | 0.00696 | 0.00000 | 0.00000 | 0.99304 | 0.00000 |
| **174** | 0.00000 | 0.00000 | 0.00649 | 0.00000 | 0.00000 | 0.99351 | 0.00000 |
| **175** | 0.00000 | 0.00000 | 0.02037 | 0.00000 | 0.00000 | 0.97963 | 0.00000 |
| **176** | 0.00000 | 0.00000 | 0.00750 | 0.00000 | 0.00000 | 0.99248 | 0.00002 |
| **177** | 0.00000 | 0.00000 | 0.00581 | 0.00051 | 0.00000 | 0.99368 | 0.00000 |
| **178** | 0.00000 | 0.00000 | 0.00835 | 0.00000 | 0.00000 | 0.99165 | 0.00000 |
| **179** | 0.00000 | 0.00000 | 0.01142 | 0.00000 | 0.00000 | 0.98858 | 0.00000 |
| **180** | 0.00000 | 0.00000 | 0.00251 | 0.00000 | 0.00000 | 0.99741 | 0.00008 |
| **181** | 0.00000 | 0.00000 | 0.04015 | 0.00000 | 0.00000 | 0.95985 | 0.00000 |
| **182** | 0.00000 | 0.00000 | 0.00000 | 0.98684 | 0.01128 | 0.00188 | 0.00000 |
| **183** | 0.00000 | 0.00000 | 0.00000 | 0.99964 | 0.00000 | 0.00036 | 0.00000 |
| **184** | 0.00000 | 0.00000 | 0.01253 | 0.93567 | 0.00053 | 0.05128 | 0.00000 |
| **185** | 0.00000 | 0.00000 | 0.00840 | 0.98922 | 0.00000 | 0.00237 | 0.00000 |
| **186** | 0.00000 | 0.00000 | 0.00000 | 0.00000 | 0.00000 | 1.00000 | 0.00000 |
| **187** | 0.00000 | 0.00000 | 0.00000 | 0.00000 | 0.00000 | 1.00000 | 0.00000 |
| **188** | 0.00000 | 0.00000 | 0.00000 | 0.00000 | 0.00000 | 1.00000 | 0.00000 |
| **189** | 0.00000 | 0.00000 | 0.00000 | 0.00000 | 0.00000 | 1.00000 | 0.00000 |
| **190** | 0.00000 | 0.00000 | 0.00000 | 0.00000 | 0.00000 | 1.00000 | 0.00000 |
| **191** | 0.00000 | 0.00000 | 0.00000 | 0.00000 | 0.00000 | 1.00000 | 0.00000 |
| **192** | 0.00000 | 0.00000 | 0.00000 | 0.00000 | 0.00000 | 1.00000 | 0.00000 |
| **193** | 0.00000 | 0.00000 | 0.00000 | 0.00000 | 0.00000 | 1.00000 | 0.00000 |
| **194** | 0.00000 | 0.00000 | 0.00000 | 0.00000 | 0.00000 | 1.00000 | 0.00000 |
| **195** | 0.00000 | 0.00000 | 0.00000 | 0.00000 | 0.00000 | 1.00000 | 0.00000 |
| **196** | 0.00000 | 0.00000 | 0.00000 | 0.00000 | 0.00001 | 0.99999 | 0.00000 |
| **197** | 0.00000 | 0.00000 | 0.00000 | 0.00000 | 0.00000 | 1.00000 | 0.00000 |
| **198** | 0.00000 | 0.00000 | 0.00000 | 0.00000 | 0.00000 | 1.00000 | 0.00000 |
| **199** | 0.00000 | 0.00000 | 0.00000 | 0.00000 | 0.00000 | 1.00000 | 0.00000 |
| **200** | 0.00000 | 0.00000 | 0.00000 | 0.00000 | 0.00324 | 0.99676 | 0.00000 |
| **201** | 0.00000 | 0.00000 | 0.00000 | 0.00000 | 0.00000 | 1.00000 | 0.00000 |
| **202** | 0.00000 | 0.00000 | 0.00000 | 0.00000 | 0.00000 | 1.00000 | 0.00000 |
| **203** | 0.00000 | 0.00000 | 0.00000 | 0.00000 | 0.00000 | 1.00000 | 0.00000 |
| **204** | 0.00000 | 0.00000 | 0.00000 | 0.00000 | 0.00000 | 1.00000 | 0.00000 |
| **205** | 0.00000 | 0.00000 | 0.00000 | 0.00000 | 0.00000 | 1.00000 | 0.00000 |
| **206** | 0.00000 | 0.00000 | 0.00000 | 0.00000 | 0.00000 | 1.00000 | 0.00000 |
| **207** | 0.00000 | 0.00000 | 0.00000 | 0.00000 | 0.00000 | 1.00000 | 0.00000 |
| **208** | 0.00000 | 0.00000 | 0.00000 | 0.00000 | 0.00000 | 1.00000 | 0.00000 |
| **209** | 0.00000 | 0.00000 | 0.00000 | 0.00000 | 0.00000 | 1.00000 | 0.00000 |
| **210** | 0.00000 | 0.00000 | 0.00000 | 0.00000 | 0.00000 | 1.00000 | 0.00000 |
| **211** | 0.00000 | 0.00000 | 0.00000 | 0.00000 | 0.00000 | 1.00000 | 0.00000 |
| **212** | 0.00000 | 0.00000 | 0.00000 | 0.00000 | 0.00000 | 1.00000 | 0.00000 |
| **213** | 0.00000 | 0.00000 | 0.00000 | 0.00000 | 0.00000 | 1.00000 | 0.00000 |
| **214** | 0.00000 | 0.00000 | 0.00000 | 0.00000 | 0.00000 | 1.00000 | 0.00000 |
| **215** | 0.00000 | 0.00000 | 0.00000 | 0.00000 | 0.00000 | 1.00000 | 0.00000 |

**Table S9** Proportion on ancestry for individuals of *Cornus kousa* subsp. *chinensis*, conditioned to climatic variables, inferred by Bayesian analysis of DNA sequence data for current and RCP45 climatic conditions

|  | **Current Inferred Cluster** | | |  | **RCP45 Inferred Cluster** | | |
| --- | --- | --- | --- | --- | --- | --- | --- |
| **Individual** | **01** | **02** | **03** | **Individual** | **01** | **02** | **03** |
| **1** | 0.08549 | 0.91449 | 0.00003 | **1** | 0.18846 | 0.81154 | 0.00000 |
| **2** | 0.04076 | 0.95920 | 0.00004 | **2** | 0.93289 | 0.06711 | 0.00000 |
| **3** | 0.16408 | 0.83585 | 0.00007 | **3** | 0.49478 | 0.50522 | 0.00000 |
| **4** | 0.02338 | 0.97658 | 0.00004 | **4** | 0.82915 | 0.17085 | 0.00000 |
| **5** | 0.99780 | 0.00214 | 0.00007 | **5** | 0.00406 | 0.00000 | 0.99594 |
| **6** | 0.01573 | 0.98418 | 0.00009 | **6** | 1.00000 | 0.00000 | 0.00000 |
| **7** | 0.53450 | 0.46549 | 0.00001 | **7** | 1.00000 | 0.00000 | 0.00000 |
| **8** | 0.95527 | 0.04451 | 0.00022 | **8** | 0.00000 | 0.00000 | 1.00000 |
| **9** | 0.99681 | 0.00297 | 0.00022 | **9** | 1.00000 | 0.00000 | 0.00000 |
| **10** | 0.77562 | 0.22423 | 0.00015 | **10** | 0.00000 | 0.00000 | 1.00000 |
| **11** | 0.04061 | 0.95923 | 0.00016 | **11** | 0.00001 | 0.00000 | 0.99999 |
| **12** | 0.01064 | 0.98927 | 0.00009 | **12** | 0.00000 | 0.00000 | 1.00000 |
| **13** | 0.10234 | 0.89764 | 0.00003 | **13** | 0.11391 | 0.88454 | 0.00155 |
| **14** | 0.01684 | 0.98314 | 0.00003 | **14** | 0.00050 | 0.99950 | 0.00000 |
| **15** | 0.07355 | 0.92643 | 0.00001 | **15** | 0.00001 | 0.99999 | 0.00000 |
| **16** | 0.05992 | 0.94003 | 0.00005 | **16** | 0.00000 | 1.00000 | 0.00000 |
| **17** | 0.01122 | 0.98873 | 0.00005 | **17** | 0.00000 | 1.00000 | 0.00000 |
| **18** | 0.07826 | 0.92166 | 0.00008 | **18** | 0.00346 | 0.99612 | 0.00042 |
| **19** | 0.37338 | 0.62657 | 0.00005 | **19** | 0.99990 | 0.00010 | 0.00000 |
| **20** | 0.26874 | 0.73120 | 0.00005 | **20** | 0.00079 | 0.99921 | 0.00000 |
| **21** | 0.18164 | 0.81836 | 0.00000 | **21** | 0.03523 | 0.96477 | 0.00000 |
| **22** | 0.23899 | 0.76097 | 0.00004 | **22** | 0.00000 | 1.00000 | 0.00000 |
| **23** | 0.11865 | 0.88131 | 0.00004 | **23** | 0.00533 | 0.99467 | 0.00000 |
| **24** | 0.09919 | 0.90073 | 0.00008 | **24** | 0.28323 | 0.71677 | 0.00000 |
| **25** | 0.25831 | 0.74164 | 0.00005 | **25** | 0.27344 | 0.72656 | 0.00000 |
| **26** | 0.32382 | 0.67601 | 0.00016 | **26** | 0.89823 | 0.10177 | 0.00000 |
| **27** | 0.11080 | 0.88916 | 0.00004 | **27** | 0.99306 | 0.00694 | 0.00000 |
| **28** | 0.99001 | 0.00989 | 0.00009 | **28** | 1.00000 | 0.00000 | 0.00000 |
| **29** | 0.98291 | 0.01704 | 0.00005 | **29** | 1.00000 | 0.00000 | 0.00000 |
| **30** | 0.99396 | 0.00574 | 0.00030 | **30** | 0.99998 | 0.00000 | 0.00002 |
| **31** | 0.98705 | 0.01289 | 0.00005 | **31** | 1.00000 | 0.00000 | 0.00000 |
| **32** | 0.96885 | 0.03108 | 0.00007 | **32** | 1.00000 | 0.00000 | 0.00000 |
| **33** | 0.82826 | 0.17164 | 0.00011 | **33** | 1.00000 | 0.00000 | 0.00000 |
| **34** | 0.92064 | 0.07922 | 0.00015 | **34** | 1.00000 | 0.00000 | 0.00000 |
| **35** | 0.96607 | 0.03381 | 0.00012 | **35** | 1.00000 | 0.00000 | 0.00000 |
| **36** | 0.97397 | 0.02597 | 0.00005 | **36** | 1.00000 | 0.00000 | 0.00000 |
| **37** | 0.94977 | 0.05009 | 0.00014 | **37** | 1.00000 | 0.00000 | 0.00000 |
| **38** | 0.97205 | 0.02791 | 0.00004 | **38** | 1.00000 | 0.00000 | 0.00000 |
| **39** | 0.91943 | 0.08046 | 0.00011 | **39** | 1.00000 | 0.00000 | 0.00000 |
| **40** | 0.97142 | 0.02843 | 0.00015 | **40** | 1.00000 | 0.00000 | 0.00000 |
| **41** | 0.93996 | 0.05992 | 0.00012 | **41** | 0.98073 | 0.01927 | 0.00000 |
| **42** | 0.94068 | 0.05932 | 0.00000 | **42** | 1.00000 | 0.00000 | 0.00000 |
| **43** | 0.98264 | 0.01732 | 0.00004 | **43** | 1.00000 | 0.00000 | 0.00000 |
| **44** | 0.88168 | 0.11827 | 0.00005 | **44** | 0.99999 | 0.00001 | 0.00000 |
| **45** | 0.96003 | 0.03992 | 0.00005 | **45** | 0.99998 | 0.00002 | 0.00000 |
| **46** | 0.97007 | 0.02992 | 0.00001 | **46** | 1.00000 | 0.00000 | 0.00000 |
| **47** | 0.04738 | 0.95259 | 0.00003 | **47** | 0.10951 | 0.89049 | 0.00000 |
| **48** | 0.10112 | 0.89885 | 0.00003 | **48** | 0.06307 | 0.93693 | 0.00000 |
| **49** | 0.10180 | 0.89820 | 0.00000 | **49** | 0.59197 | 0.40803 | 0.00000 |
| **50** | 0.12346 | 0.87651 | 0.00003 | **50** | 0.26097 | 0.73903 | 0.00000 |
| **51** | 0.03292 | 0.96704 | 0.00004 | **51** | 0.02681 | 0.97319 | 0.00000 |
| **52** | 0.06062 | 0.93935 | 0.00003 | **52** | 0.30426 | 0.69574 | 0.00000 |
| **53** | 0.08274 | 0.91724 | 0.00001 | **53** | 0.25880 | 0.73232 | 0.00888 |
| **54** | 0.08404 | 0.91588 | 0.00008 | **54** | 0.39308 | 0.60692 | 0.00000 |
| **55** | 0.06711 | 0.93289 | 0.00000 | **55** | 0.00002 | 0.99998 | 0.00000 |
| **56** | 0.05384 | 0.94614 | 0.00003 | **56** | 0.04253 | 0.95747 | 0.00000 |
| **57** | 0.04400 | 0.95599 | 0.00001 | **57** | 0.02708 | 0.97292 | 0.00000 |
| **58** | 0.04176 | 0.95823 | 0.00001 | **58** | 0.25449 | 0.74551 | 0.00000 |
| **59** | 0.23315 | 0.76678 | 0.00007 | **59** | 0.04645 | 0.95355 | 0.00000 |
| **60** | 0.27919 | 0.72078 | 0.00003 | **60** | 0.00001 | 0.99999 | 0.00000 |
| **61** | 0.10927 | 0.89070 | 0.00003 | **61** | 1.00000 | 0.00000 | 0.00000 |
| **62** | 0.01484 | 0.98499 | 0.00018 | **62** | 0.00001 | 0.99999 | 0.00000 |
| **63** | 0.20085 | 0.79893 | 0.00022 | **63** | 0.00000 | 1.00000 | 0.00000 |
| **64** | 0.41950 | 0.58046 | 0.00004 | **64** | 0.99995 | 0.00000 | 0.00005 |
| **65** | 0.12595 | 0.87396 | 0.00009 | **65** | 0.00000 | 1.00000 | 0.00000 |
| **66** | 0.37458 | 0.62541 | 0.00001 | **66** | 1.00000 | 0.00000 | 0.00000 |
| **67** | 0.40636 | 0.59361 | 0.00003 | **67** | 0.00000 | 1.00000 | 0.00000 |
| **68** | 0.69566 | 0.30423 | 0.00011 | **68** | 0.01788 | 0.98212 | 0.00000 |
| **69** | 0.36215 | 0.63776 | 0.00009 | **69** | 0.00010 | 0.99990 | 0.00000 |
| **70** | 0.80414 | 0.19577 | 0.00009 | **70** | 0.99699 | 0.00301 | 0.00000 |
| **71** | 0.58709 | 0.25081 | 0.16209 | **71** | 1.00000 | 0.00000 | 0.00000 |
| **72** | 0.84635 | 0.15358 | 0.00007 | **72** | 0.99936 | 0.00063 | 0.00000 |
| **73** | 0.72685 | 0.27295 | 0.00020 | **73** | 1.00000 | 0.00000 | 0.00000 |
| **74** | 0.86014 | 0.13955 | 0.00031 | **74** | 0.41764 | 0.58236 | 0.00000 |
| **75** | 0.80615 | 0.19365 | 0.00020 | **75** | 1.00000 | 0.00000 | 0.00000 |
| **76** | 0.96777 | 0.03215 | 0.00008 | **76** | 1.00000 | 0.00000 | 0.00000 |
| **77** | 0.99336 | 0.00661 | 0.00003 | **77** | 0.99994 | 0.00000 | 0.00006 |
| **78** | 0.94246 | 0.05746 | 0.00008 | **78** | 1.00000 | 0.00000 | 0.00000 |
| **79** | 0.98086 | 0.01905 | 0.00008 | **79** | 1.00000 | 0.00000 | 0.00000 |
| **80** | 0.25896 | 0.01497 | 0.72607 | **80** | 1.00000 | 0.00000 | 0.00000 |
| **81** | 0.99488 | 0.00507 | 0.00005 | **81** | 1.00000 | 0.00000 | 0.00000 |
| **82** | 0.97223 | 0.02772 | 0.00005 | **82** | 1.00000 | 0.00000 | 0.00000 |
| **83** | 0.95331 | 0.04665 | 0.00004 | **83** | 0.99993 | 0.00007 | 0.00000 |
| **84** | 0.98635 | 0.01361 | 0.00004 | **84** | 1.00000 | 0.00000 | 0.00000 |
| **85** | 0.95861 | 0.04131 | 0.00008 | **85** | 0.97012 | 0.00000 | 0.02988 |
| **86** | 0.97134 | 0.02859 | 0.00007 | **86** | 1.00000 | 0.00000 | 0.00000 |
| **87** | 0.95442 | 0.04546 | 0.00012 | **87** | 1.00000 | 0.00000 | 0.00000 |
| **88** | 0.98565 | 0.01432 | 0.00003 | **88** | 1.00000 | 0.00000 | 0.00000 |
| **89** | 0.98528 | 0.01458 | 0.00014 | **89** | 1.00000 | 0.00000 | 0.00000 |
| **90** | 0.96541 | 0.03453 | 0.00007 | **90** | 1.00000 | 0.00000 | 0.00000 |
| **91** | 0.00819 | 0.99178 | 0.00003 | **91** | 0.00000 | 1.00000 | 0.00000 |
| **92** | 0.00189 | 0.99805 | 0.00005 | **92** | 0.00305 | 0.99695 | 0.00000 |
| **93** | 0.03504 | 0.96493 | 0.00003 | **93** | 0.00001 | 0.99999 | 0.00000 |
| **94** | 0.20885 | 0.79112 | 0.00003 | **94** | 0.00141 | 0.99859 | 0.00000 |
| **95** | 0.00420 | 0.99580 | 0.00000 | **95** | 0.18238 | 0.81762 | 0.00000 |
| **96** | 0.00165 | 0.99832 | 0.00003 | **96** | 0.00000 | 1.00000 | 0.00000 |
| **97** | 0.58103 | 0.41895 | 0.00003 | **97** | 0.00000 | 1.00000 | 0.00000 |
| **98** | 0.49685 | 0.50314 | 0.00001 | **98** | 0.00000 | 1.00000 | 0.00000 |
| **99** | 0.24501 | 0.75499 | 0.00000 | **99** | 0.00000 | 1.00000 | 0.00000 |
| **100** | 0.33418 | 0.66578 | 0.00004 | **100** | 0.99713 | 0.00287 | 0.00000 |
| **101** | 0.05641 | 0.94354 | 0.00005 | **101** | 0.31583 | 0.68417 | 0.00000 |
| **102** | 0.25445 | 0.74553 | 0.00003 | **102** | 0.00001 | 0.99999 | 0.00000 |
| **103** | 0.13608 | 0.86388 | 0.00004 | **103** | 0.99421 | 0.00579 | 0.00000 |
| **104** | 0.13589 | 0.86411 | 0.00000 | **104** | 0.06741 | 0.93259 | 0.00000 |
| **105** | 0.34232 | 0.65761 | 0.00007 | **105** | 0.00549 | 0.99451 | 0.00000 |
| **106** | 0.09957 | 0.90032 | 0.00011 | **106** | 0.46103 | 0.53897 | 0.00000 |
| **107** | 0.21876 | 0.78119 | 0.00005 | **107** | 0.00058 | 0.99942 | 0.00000 |
| **108** | 0.09772 | 0.90227 | 0.00001 | **108** | 0.00000 | 1.00000 | 0.00000 |
| **109** | 0.14708 | 0.32616 | 0.52676 | **109** | 0.00045 | 0.99955 | 0.00000 |
| **110** | 0.12396 | 0.87596 | 0.00008 | **110** | 0.00856 | 0.99143 | 0.00000 |
| **111** | 0.13295 | 0.86703 | 0.00003 | **111** | 0.00578 | 0.99422 | 0.00000 |
| **112** | 0.12495 | 0.87497 | 0.00008 | **112** | 0.04228 | 0.95772 | 0.00000 |
| **113** | 0.06311 | 0.93685 | 0.00004 | **113** | 0.49687 | 0.50313 | 0.00000 |
| **114** | 0.16688 | 0.83301 | 0.00011 | **114** | 0.00001 | 0.99999 | 0.00000 |
| **115** | 0.11018 | 0.88980 | 0.00003 | **115** | 0.00000 | 1.00000 | 0.00000 |
| **116** | 0.05400 | 0.94592 | 0.00008 | **116** | 0.00001 | 0.99999 | 0.00000 |
| **117** | 0.12016 | 0.87969 | 0.00015 | **117** | 0.00039 | 0.99961 | 0.00000 |
| **118** | 0.12118 | 0.87882 | 0.00000 | **118** | 0.00000 | 1.00000 | 0.00000 |
| **119** | 0.04699 | 0.95295 | 0.00007 | **119** | 0.02921 | 0.97079 | 0.00000 |
| **120** | 0.01392 | 0.98600 | 0.00008 | **120** | 0.00908 | 0.99092 | 0.00000 |
| **121** | 0.16542 | 0.83453 | 0.00005 | **121** | 0.00067 | 0.99933 | 0.00000 |
| **122** | 0.20868 | 0.79118 | 0.00015 | **122** | 0.00019 | 0.99981 | 0.00000 |
| **123** | 0.04088 | 0.95909 | 0.00003 | **123** | 0.00001 | 0.99999 | 0.00000 |
| **124** | 0.03414 | 0.96580 | 0.00007 | **124** | 0.00004 | 0.99996 | 0.00000 |
| **125** | 0.11686 | 0.88305 | 0.00008 | **125** | 0.00000 | 1.00000 | 0.00000 |
| **126** | 0.07307 | 0.92676 | 0.00018 | **126** | 0.00000 | 1.00000 | 0.00000 |
| **127** | 0.72462 | 0.27528 | 0.00009 | **127** | 0.01059 | 0.98941 | 0.00000 |
| **128** | 0.29082 | 0.70914 | 0.00004 | **128** | 0.00328 | 0.99672 | 0.00000 |
| **129** | 0.50741 | 0.49253 | 0.00007 | **129** | 0.00000 | 1.00000 | 0.00000 |
| **130** | 0.00512 | 0.99478 | 0.00009 | **130** | 0.01061 | 0.98939 | 0.00000 |
| **131** | 0.20604 | 0.79393 | 0.00003 | **131** | 0.14372 | 0.85628 | 0.00000 |
| **132** | 0.16666 | 0.83328 | 0.00005 | **132** | 0.00000 | 1.00000 | 0.00000 |
| **133** | 0.04401 | 0.95597 | 0.00001 | **133** | 0.00000 | 1.00000 | 0.00000 |
| **134** | 0.06805 | 0.93193 | 0.00001 | **134** | 0.00000 | 1.00000 | 0.00000 |
| **135** | 0.08495 | 0.91504 | 0.00001 | **135** | 0.00000 | 1.00000 | 0.00000 |
| **136** | 0.04761 | 0.95235 | 0.00004 | **136** | 0.00000 | 1.00000 | 0.00000 |
| **137** | 0.01982 | 0.98005 | 0.00012 | **137** | 0.00001 | 0.99999 | 0.00000 |
| **138** | 0.34088 | 0.65909 | 0.00003 | **138** | 0.00000 | 1.00000 | 0.00000 |
| **139** | 0.31743 | 0.68251 | 0.00005 | **139** | 0.00000 | 1.00000 | 0.00000 |
| **140** | 0.00007 | 0.00009 | 0.99984 | **140** | 0.00000 | 0.00000 | 1.00000 |
| **141** | 0.00022 | 0.00008 | 0.99970 | **141** | 1.00000 | 0.00000 | 0.00000 |
| **142** | 0.00031 | 0.00005 | 0.99964 | **142** | 0.99999 | 0.00000 | 0.00001 |
| **143** | 0.00001 | 0.00003 | 0.99996 | **143** | 1.00000 | 0.00000 | 0.00000 |
| **144** | 0.95664 | 0.04323 | 0.00014 | **144** | 1.00000 | 0.00000 | 0.00000 |
| **145** | 0.94762 | 0.05231 | 0.00007 | **145** | 0.99979 | 0.00021 | 0.00000 |
| **146** | 0.97192 | 0.02807 | 0.00001 | **146** | 1.00000 | 0.00000 | 0.00000 |
| **147** | 0.92573 | 0.07416 | 0.00011 | **147** | 1.00000 | 0.00000 | 0.00000 |
| **148** | 0.97926 | 0.02058 | 0.00016 | **148** | 1.00000 | 0.00000 | 0.00000 |
| **149** | 0.94039 | 0.05961 | 0.00000 | **149** | 1.00000 | 0.00000 | 0.00000 |
| **150** | 0.97574 | 0.02419 | 0.00007 | **150** | 1.00000 | 0.00000 | 0.00000 |
| **151** | 0.96503 | 0.03495 | 0.00003 | **151** | 1.00000 | 0.00000 | 0.00000 |
| **152** | 0.98166 | 0.01822 | 0.00012 | **152** | 1.00000 | 0.00000 | 0.00000 |
| **153** | 0.96514 | 0.03474 | 0.00012 | **153** | 1.00000 | 0.00000 | 0.00000 |
| **154** | 0.23709 | 0.76282 | 0.00008 | **154** | 1.00000 | 0.00000 | 0.00000 |
| **155** | 0.44572 | 0.55423 | 0.00005 | **155** | 1.00000 | 0.00000 | 0.00000 |
| **156** | 0.32765 | 0.67231 | 0.00004 | **156** | 1.00000 | 0.00000 | 0.00000 |
| **157** | 0.11691 | 0.88300 | 0.00009 | **157** | 1.00000 | 0.00000 | 0.00000 |
| **158** | 0.49081 | 0.50903 | 0.00016 | **158** | 1.00000 | 0.00000 | 0.00000 |
| **159** | 0.37296 | 0.62696 | 0.00008 | **159** | 1.00000 | 0.00000 | 0.00000 |
| **160** | 0.19282 | 0.80708 | 0.00009 | **160** | 0.00044 | 0.99956 | 0.00000 |
| **161** | 0.79715 | 0.20278 | 0.00007 | **161** | 0.95645 | 0.04355 | 0.00000 |
| **162** | 0.15080 | 0.84911 | 0.00009 | **162** | 0.96651 | 0.03349 | 0.00000 |
| **163** | 0.07177 | 0.92823 | 0.00000 | **163** | 1.00000 | 0.00000 | 0.00000 |
| **164** | 0.17681 | 0.82307 | 0.00012 | **164** | 0.99974 | 0.00026 | 0.00000 |
| **165** | 0.12684 | 0.87299 | 0.00018 | **165** | 0.98288 | 0.01712 | 0.00000 |
| **166** | 0.07153 | 0.92841 | 0.00007 | **166** | 0.68932 | 0.31068 | 0.00000 |
| **167** | 0.08134 | 0.91857 | 0.00009 | **167** | 0.99833 | 0.00167 | 0.00000 |
| **168** | 0.00218 | 0.99777 | 0.00005 | **168** | 0.00008 | 0.99992 | 0.00000 |
| **169** | 0.13100 | 0.86892 | 0.00008 | **169** | 0.01158 | 0.98842 | 0.00000 |
| **170** | 0.37557 | 0.62443 | 0.00000 | **170** | 0.16974 | 0.83026 | 0.00000 |
| **171** | 0.40789 | 0.59211 | 0.00000 | **171** | 0.00000 | 1.00000 | 0.00000 |
| **172** | 0.26818 | 0.73173 | 0.00009 | **172** | 0.06461 | 0.93539 | 0.00000 |
| **173** | 0.01726 | 0.98272 | 0.00003 | **173** | 0.11432 | 0.88568 | 0.00000 |
| **174** | 0.34482 | 0.65512 | 0.00005 | **174** | 0.00595 | 0.99405 | 0.00000 |
| **175** | 0.00186 | 0.99814 | 0.00000 | **175** | 0.13660 | 0.86340 | 0.00000 |
| **176** | 0.25797 | 0.74201 | 0.00001 | **176** | 0.00000 | 0.99998 | 0.00002 |
| **177** | 0.01372 | 0.98627 | 0.00001 | **177** | 0.00005 | 0.99995 | 0.00000 |
| **178** | 0.06939 | 0.93057 | 0.00004 | **178** | 0.00000 | 1.00000 | 0.00000 |
| **179** | 0.03453 | 0.96543 | 0.00004 | **179** | 0.00001 | 0.99999 | 0.00000 |
| **180** | 0.00384 | 0.99609 | 0.00007 | **180** | 0.00000 | 1.00000 | 0.00000 |
| **181** | 0.01511 | 0.98484 | 0.00005 | **181** | 0.00051 | 0.99949 | 0.00000 |
| **182** | 0.02228 | 0.97766 | 0.00005 | **182** | 0.00001 | 0.99999 | 0.00000 |
| **183** | 0.27620 | 0.72372 | 0.00008 | **183** | 0.00001 | 0.99999 | 0.00000 |
| **184** | 0.07816 | 0.92182 | 0.00001 | **184** | 0.22774 | 0.77226 | 0.00000 |
| **185** | 0.08439 | 0.91559 | 0.00001 | **185** | 0.12490 | 0.87510 | 0.00000 |
| **186** | 0.10849 | 0.89142 | 0.00009 | **186** | 0.00032 | 0.99968 | 0.00000 |

**Table S10** Proportion on ancestry for individuals of *Cornus kousa* subsp. *chinensis*, conditioned to environmental variables, inferred by Bayesian analysis of DNA sequence data for RCP85 climatic conditions.

|  | **Inferred Cluster** | | |  | **Inferred Cluster** | | |
| --- | --- | --- | --- | --- | --- | --- | --- |
| **Individual** | **01** | **02** | **03** | **Individual** | **01** | **02** | **03** |
| **1** | 0.99176 | 0.00824 | 0.00000 | **94** | 0.61072 | 0.38928 | 0.00000 |
| **2** | 1.00000 | 0.00000 | 0.00000 | **95** | 1.00000 | 0.00000 | 0.00000 |
| **3** | 1.00000 | 0.00000 | 0.00000 | **96** | 1.00000 | 0.00000 | 0.00000 |
| **4** | 1.00000 | 0.00000 | 0.00000 | **97** | 0.00000 | 0.00000 | 1.00000 |
| **5** | 1.00000 | 0.00000 | 0.00000 | **98** | 0.00000 | 0.00000 | 1.00000 |
| **6** | 1.00000 | 0.00000 | 0.00000 | **99** | 1.00000 | 0.00000 | 0.00000 |
| **7** | 1.00000 | 0.00000 | 0.00000 | **100** | 0.00000 | 1.00000 | 0.00000 |
| **8** | 1.00000 | 0.00000 | 0.00000 | **101** | 0.44861 | 0.55139 | 0.00000 |
| **9** | 0.00000 | 0.00000 | 1.00000 | **102** | 0.01273 | 0.98727 | 0.00000 |
| **10** | 0.99998 | 0.00002 | 0.00000 | **103** | 0.00003 | 0.99997 | 0.00000 |
| **11** | 1.00000 | 0.00000 | 0.00000 | **104** | 0.01321 | 0.98679 | 0.00000 |
| **12** | 0.99878 | 0.00122 | 0.00000 | **105** | 0.07822 | 0.92178 | 0.00000 |
| **13** | 1.00000 | 0.00000 | 0.00000 | **106** | 0.51626 | 0.48374 | 0.00000 |
| **14** | 1.00000 | 0.00000 | 0.00000 | **107** | 0.04384 | 0.95616 | 0.00000 |
| **15** | 1.00000 | 0.00000 | 0.00000 | **108** | 0.11768 | 0.88232 | 0.00000 |
| **16** | 1.00000 | 0.00000 | 0.00000 | **109** | 0.25761 | 0.74239 | 0.00000 |
| **17** | 1.00000 | 0.00000 | 0.00000 | **110** | 0.00672 | 0.99328 | 0.00000 |
| **18** | 1.00000 | 0.00000 | 0.00000 | **111** | 0.00004 | 0.99968 | 0.00028 |
| **19** | 0.98821 | 0.01179 | 0.00000 | **112** | 0.00308 | 0.99692 | 0.00000 |
| **20** | 0.13105 | 0.86895 | 0.00000 | **113** | 0.00742 | 0.99258 | 0.00000 |
| **21** | 0.97663 | 0.00964 | 0.01373 | **114** | 0.84623 | 0.15377 | 0.00000 |
| **22** | 0.97935 | 0.02065 | 0.00000 | **115** | 0.04027 | 0.95973 | 0.00000 |
| **23** | 0.07983 | 0.92017 | 0.00000 | **116** | 0.09852 | 0.90148 | 0.00000 |
| **24** | 0.90183 | 0.09817 | 0.00000 | **117** | 1.00000 | 0.00000 | 0.00000 |
| **25** | 0.75364 | 0.24636 | 0.00000 | **118** | 1.00000 | 0.00000 | 0.00000 |
| **26** | 1.00000 | 0.00000 | 0.00000 | **119** | 0.99900 | 0.00100 | 0.00000 |
| **27** | 0.94105 | 0.05895 | 0.00000 | **120** | 1.00000 | 0.00000 | 0.00000 |
| **28** | 1.00000 | 0.00000 | 0.00000 | **121** | 0.99400 | 0.00000 | 0.00600 |
| **29** | 1.00000 | 0.00000 | 0.00000 | **122** | 0.01250 | 0.00000 | 0.98750 |
| **30** | 1.00000 | 0.00000 | 0.00000 | **123** | 1.00000 | 0.00000 | 0.00000 |
| **31** | 1.00000 | 0.00000 | 0.00000 | **124** | 1.00000 | 0.00000 | 0.00000 |
| **32** | 1.00000 | 0.00000 | 0.00000 | **125** | 1.00000 | 0.00000 | 0.00000 |
| **33** | 1.00000 | 0.00000 | 0.00000 | **126** | 0.99950 | 0.00050 | 0.00000 |
| **34** | 1.00000 | 0.00000 | 0.00000 | **127** | 0.99360 | 0.00640 | 0.00000 |
| **35** | 1.00000 | 0.00000 | 0.00000 | **128** | 0.58564 | 0.41436 | 0.00000 |
| **36** | 1.00000 | 0.00000 | 0.00000 | **129** | 0.99997 | 0.00003 | 0.00000 |
| **37** | 1.00000 | 0.00000 | 0.00000 | **130** | 0.00000 | 1.00000 | 0.00000 |
| **38** | 0.92466 | 0.07534 | 0.00000 | **131** | 0.99992 | 0.00008 | 0.00000 |
| **39** | 1.00000 | 0.00000 | 0.00000 | **132** | 0.02362 | 0.97638 | 0.00000 |
| **40** | 1.00000 | 0.00000 | 0.00000 | **133** | 1.00000 | 0.00000 | 0.00000 |
| **41** | 1.00000 | 0.00000 | 0.00000 | **134** | 1.00000 | 0.00000 | 0.00000 |
| **42** | 1.00000 | 0.00000 | 0.00000 | **135** | 0.99219 | 0.00781 | 0.00000 |
| **43** | 1.00000 | 0.00000 | 0.00000 | **136** | 0.00001 | 0.99999 | 0.00000 |
| **44** | 1.00000 | 0.00000 | 0.00000 | **137** | 0.66464 | 0.33536 | 0.00000 |
| **45** | 1.00000 | 0.00000 | 0.00000 | **138** | 0.99773 | 0.00227 | 0.00000 |
| **46** | 1.00000 | 0.00000 | 0.00000 | **139** | 0.99969 | 0.00031 | 0.00000 |
| **47** | 0.99999 | 0.00001 | 0.00000 | **140** | 1.00000 | 0.00000 | 0.00000 |
| **48** | 1.00000 | 0.00000 | 0.00000 | **141** | 0.00183 | 0.00000 | 0.99817 |
| **49** | 0.61111 | 0.38889 | 0.00000 | **142** | 1.00000 | 0.00000 | 0.00000 |
| **50** | 1.00000 | 0.00000 | 0.00000 | **143** | 1.00000 | 0.00000 | 0.00000 |
| **51** | 0.99979 | 0.00021 | 0.00000 | **144** | 1.00000 | 0.00000 | 0.00000 |
| **52** | 0.75779 | 0.24221 | 0.00000 | **145** | 1.00000 | 0.00000 | 0.00000 |
| **53** | 0.99913 | 0.00087 | 0.00000 | **146** | 0.99758 | 0.00000 | 0.00242 |
| **54** | 0.93310 | 0.06690 | 0.00000 | **147** | 1.00000 | 0.00000 | 0.00000 |
| **55** | 0.98941 | 0.01059 | 0.00000 | **148** | 1.00000 | 0.00000 | 0.00000 |
| **56** | 1.00000 | 0.00000 | 0.00000 | **149** | 1.00000 | 0.00000 | 0.00000 |
| **57** | 0.98519 | 0.01481 | 0.00000 | **150** | 1.00000 | 0.00000 | 0.00000 |
| **58** | 0.86542 | 0.13458 | 0.00000 | **151** | 1.00000 | 0.00000 | 0.00000 |
| **59** | 0.00119 | 0.99881 | 0.00000 | **152** | 0.99998 | 0.00000 | 0.00002 |
| **60** | 1.00000 | 0.00000 | 0.00000 | **153** | 1.00000 | 0.00000 | 0.00000 |
| **61** | 1.00000 | 0.00000 | 0.00000 | **154** | 1.00000 | 0.00000 | 0.00000 |
| **62** | 1.00000 | 0.00000 | 0.00000 | **155** | 0.99839 | 0.00161 | 0.00000 |
| **63** | 0.99682 | 0.00316 | 0.00001 | **156** | 0.20630 | 0.00000 | 0.79370 |
| **64** | 1.00000 | 0.00000 | 0.00000 | **157** | 1.00000 | 0.00000 | 0.00000 |
| **65** | 1.00000 | 0.00000 | 0.00000 | **158** | 1.00000 | 0.00000 | 0.00000 |
| **66** | 0.99989 | 0.00000 | 0.00011 | **159** | 1.00000 | 0.00000 | 0.00000 |
| **67** | 0.00012 | 0.99988 | 0.00000 | **160** | 0.99998 | 0.00000 | 0.00002 |
| **68** | 1.00000 | 0.00000 | 0.00000 | **161** | 1.00000 | 0.00000 | 0.00000 |
| **69** | 1.00000 | 0.00000 | 0.00000 | **162** | 1.00000 | 0.00000 | 0.00000 |
| **70** | 0.99987 | 0.00013 | 0.00000 | **163** | 1.00000 | 0.00000 | 0.00000 |
| **71** | 0.99625 | 0.00375 | 0.00000 | **164** | 1.00000 | 0.00000 | 0.00000 |
| **72** | 0.97345 | 0.02653 | 0.00002 | **165** | 1.00000 | 0.00000 | 0.00000 |
| **73** | 0.95135 | 0.04637 | 0.00228 | **166** | 1.00000 | 0.00000 | 0.00000 |
| **74** | 0.94986 | 0.05014 | 0.00000 | **167** | 1.00000 | 0.00000 | 0.00000 |
| **75** | 0.55642 | 0.44358 | 0.00000 | **168** | 1.00000 | 0.00000 | 0.00000 |
| **76** | 1.00000 | 0.00000 | 0.00000 | **169** | 1.00000 | 0.00000 | 0.00000 |
| **77** | 0.97979 | 0.00000 | 0.02021 | **170** | 0.99981 | 0.00019 | 0.00000 |
| **78** | 1.00000 | 0.00000 | 0.00000 | **171** | 0.00118 | 0.99882 | 0.00000 |
| **79** | 1.00000 | 0.00000 | 0.00000 | **172** | 1.00000 | 0.00000 | 0.00000 |
| **80** | 1.00000 | 0.00000 | 0.00000 | **173** | 1.00000 | 0.00000 | 0.00000 |
| **81** | 1.00000 | 0.00000 | 0.00000 | **174** | 1.00000 | 0.00000 | 0.00000 |
| **82** | 1.00000 | 0.00000 | 0.00000 | **175** | 1.00000 | 0.00000 | 0.00000 |
| **83** | 1.00000 | 0.00000 | 0.00000 | **176** | 1.00000 | 0.00000 | 0.00000 |
| **84** | 1.00000 | 0.00000 | 0.00000 | **177** | 0.96496 | 0.00514 | 0.02990 |
| **85** | 1.00000 | 0.00000 | 0.00000 | **178** | 0.73923 | 0.26075 | 0.00002 |
| **86** | 1.00000 | 0.00000 | 0.00000 | **179** | 0.73992 | 0.26008 | 0.00000 |
| **87** | 0.91845 | 0.00000 | 0.08155 | **180** | 0.99986 | 0.00014 | 0.00000 |
| **88** | 0.90599 | 0.00000 | 0.09401 | **181** | 1.00000 | 0.00000 | 0.00000 |
| **89** | 1.00000 | 0.00000 | 0.00000 | **182** | 1.00000 | 0.00000 | 0.00000 |
| **90** | 1.00000 | 0.00000 | 0.00000 | **183** | 0.99993 | 0.00007 | 0.00000 |
| **91** | 1.00000 | 0.00000 | 0.00000 | **184** | 0.18352 | 0.81648 | 0.00000 |
| **92** | 0.99969 | 0.00031 | 0.00000 | **185** | 0.25069 | 0.74930 | 0.00000 |
| **93** | 1.00000 | 0.00000 | 0.00000 | **186** | 0.89984 | 0.10016 | 0.00000 |
